# Phosphorus lability across diverse agricultural contexts with legacy sources

**DOI:** 10.1002/jeq2.20632

**Published:** 2024-09-29

**Authors:** Zachary P. Simpson, Joshua Mott, Kyle Elkin, Anthony Buda, Joshua Faulkner, Cathleen Hapeman, Greg McCarty, Maryam Foroughi, W. Dean Hively, Kevin King, Will Osterholz, Chad Penn, Mark Williams, Lindsey Witthaus, Martin Locke, Ethan Pawlowski, Brent Dalzell, Gary Feyereisen, Christine Dolph, David Bjorneberg, Kossi Nouwakpo, Christopher W. Rogers, Isis Scott, Carl H. Bolster, Lisa Duriancik, Peter J. A. Kleinman

**Affiliations:** ^1^ USDA‐ARS, Sustainable Water Management Research Unit Stoneville Mississippi USA; ^2^ USDA‐ARS, Soil Management and Sugar Beet Research Unit Fort Collins Colorado USA; ^3^ USDA‐ARS, Pasture Systems and Watershed Management Research Unit University Park Pennsylvania USA; ^4^ Center for Sustainable Agriculture, University of Vermont Burlington Vermont USA; ^5^ USDA‐ARS, Hydrology and Remote Sensing Laboratory (HRSL) Beltsville Maryland USA; ^6^ College of Agriculture and Natural Resources University of Maryland College Park Maryland USA; ^7^ USGS, Lower Mississippi‐Gulf Science Center Beltsville Maryland USA; ^8^ USDA‐ARS, Soil Drainage Research Unit Columbus Ohio USA; ^9^ USDA‐ARS, National Soil Erosion Research Laboratory West Lafayette Indiana USA; ^10^ USDA‐ARS, National Sedimentation Laboratory Oxford Mississippi USA; ^11^ USDA‐ARS, Soil and Water Management Research Unit St. Paul Minnesota USA; ^12^ Department of Ecology, Evolution and Behavior University of Minnesota St. Paul Minnesota USA; ^13^ USDA‐ARS, Northwest Irrigation and Soils Research Unit Kimberly Idaho USA; ^14^ Department of Biological and Agricultural Engineering Kansas State University Manhattan Kansas USA; ^15^ USDA‐ARS, Food Animal Environmental Systems Research Unit Bowling Green Kentucky USA; ^16^ USDA‐NRCS, Resource Assessment Branch, Conservation Effects Assessment Project Beltsville Maryland USA

## Abstract

The buffering of phosphorus (P) in the landscape delays management outcomes for water quality. If stored in labile form (readily exchangeable and bioavailable), P may readily pollute waters. We studied labile P and its intensity for >600 soils and sediments across seven study locations in the United States. Stocks of labile P were large enough to sustain high P losses for decades, indicating the transport‐limited regime typical of legacy P. Sediments were commonly more P‐sorptive than nearby soils. Soils in the top 5 cm had 1.3–3.0 times more labile P than soils at 5–15 cm. Stratification in soil test P and total P was, however, less consistent. As P exchange via sorption processes follows the difference in intensities between soil/sediment surface and solution, we built a model for the equilibrium phosphate concentration at net zero sorption (EPC_0_) as a function of labile P (quantity) and buffer capacity. Despite widely varying properties across sites, the model generalized well for all soils and sediments: EPC_0_ increased sharply with more labile P and to greater degree when buffer capacity was low or sorption sites were likely more saturated. This quantity–intensity–capacity relationship is central to the P transport models we rely on today. Our data inform the improvement of such P models, which will be necessary to predict the impacts of legacy P. Further, this work reaffirms the position of labile P as a key focus for environmental P management—a view Dr. Sharpley developed in the 1980s with fewer data and resources.

## Core Ideas

- The USDA Legacy P project analyzed >600 soils/sediments across seven participating watersheds.
- Soil labile P stored in top 1 cm at all sites are enough to potentially sustain high P losses for decades.
- Soil P at all sites was vertically stratified, particularly so for labile P, representing a risk for P loss.
- P intensity (equilibrium phosphate concentration at net zero sorption [EPC_0_]) in all soils and sediments was described through a quantity–intensity–capacity relationship.
- Data here can inform the future generations of P transport models needed to address legacy P.

**Abbreviations**
AEM: anion exchange membrane
AIC: Akaike information criterion
BWI: Bache–Williams index
DPS: degree of phosphorus saturation
EPC_0_
: equilibrium phosphate concentration at net zero sorption
EPIC: erosion productivity impact calculator
GAM: generalized additive model
OC: organic carbon
SWAT: soil and water assessment tool
WEP: water extractable phosphorus

## 1 INTRODUCTION

When it comes to phosphorus (P), we live with our actions for a long time: Sharpley et al. (2013) summarized this legacy P effect and how it will, without intervention, degrade aquatic ecosystems for decades or longer. The legacy P challenge is exacerbated by two effects: (1) Relatively little P is needed to eutrophy surface waters; and (2) P in the environment preferentially distributes among the surfaces of soils and sediments rather than in solution (Kleinman et al., 2011). The portion of total P in soils and sediments that can readily exchange with P in solution (porewater, runoff, and water column) and is readily available to microbes and plants is considered the *labile* P pool (Beckett & White, 1964; Holford, 1997; Sharpley et al., 1984). This labile P, which may be in dynamic equilibrium with more stable pools, can be large in settings emblematic of “legacy P.” As labile P can continuously pollute waters if given the opportunity, it deserves critical attention in quantifying the potential for legacy P sources to contribute to watershed P loads.

Labile P dynamics are central to understanding P fate in the environment and modern P transport modeling. Following the operational definition by Sharpley et al. (1984) (quantity of P desorbed in 24 h in presence of anion exchange resins), labile P is now key to several current simulation models (Das et al., 2019). This is largely due to the influence of the erosion productivity impact calculator (EPIC) model (Jones et al., 1984), which conceptualizes inorganic P in soil as in dynamic equilibrium between labile P, “active” inorganic P, and “stable” inorganic P pools. For example, Vadas and White (2010) discuss the lineage of this P model to the widely used soil and water assessment tool (SWAT) model. While “active” and “stable” P pools are conceptual, labile P is observable. Indeed, using soil P extractions with anion exchange resins, Sharpley and colleagues built the empirical datasets and functions underlying EPIC, which shaped models like SWAT, annual P loss estimator, and others (Radcliffe & Cabrera, 2006; Sharpley et al., 1984; Sharpley & Williams, 1990). However, to build better models for predicting legacy P effects on watershed outcomes, data are needed from additional pedoclimates, soil chemistries, and agricultural systems with varying historical inputs of P.

Previous comprehensive studies of soil and sediment P properties revealed important predictors for P lability. Using isotope exchange kinetics across 102 forest soils in France (total P of 20–1800 mg P kg^−1^), Achat et al. (2016) determined that organic carbon (OC), iron (Fe) and aluminum (Al) oxides, and clay concentration were highly predictive of soil solution P (see also similar result for peaty soils in Schoumans [2013]). In a review of 53 soils, Helfenstein et al. (2020) quantified an average turnover time of ∼1 h for the exchange of labile P with solution P. Turnover times were shorter for soils with greater clay concentration but longer for arable soils with a history of greater P applications, presumably due to a diminished P buffer capacity and larger labile P pool. The diverse 78 soils in Sharpley et al. (1984) suggested linear relationships between labile P and soil test P; however, the soil test P was comparatively moderate (e.g., median Olsen P ranged 9–19 mg P kg^−1^), which limits the applicability for settings with greater amounts of Sharpley (1995) later noted the soil‐specific relationships between runoff P and soil test P, meaning that common soil tests may not generalize easily across settings unlike P lability.

Fundamental to labile P dynamics, the quantity–intensity–capacity relationship, or P lability for short, describes how the labile P quantity and buffer capacity together determine solution P concentration, or intensity (Bache & Williams, 1971; Pierzynski et al., 2005; Sharpley, 1983). The P intensity (i.e., solution activity of P) is a strong predictor of runoff P (McDowell, Sinaj, et al., 2001) and it is supplied by the quantity of labile, or readily desorbable, P on the soil or sediment surface (Sharpley & Ahuja, 1983). McDowell and Condron (2004) and Sharpley et al. (1981) synthesized runoff P data from United States, United Kingdom, and New Zealand, resulting in a strong relationship between runoff P concentrations and soil P intensities (measured as water‐ or CaCl_2_‐extractable P). Further, they suggested that soil test P normalized to P buffering capacity was a strong predictor of runoff P. Likewise, Penn et al. (2006) found that conditioning on clay concentration (a proxy for P buffer capacity) unified relationships between runoff P and soil water‐extractable P for contrasting physiographic regions in Virginia. Such studies support the quantity–intensity–capacity hypothesis and provide the empirical basis needed to generalize to other settings lacking data. However, datasets of all three variables (e.g., Pierzynski et al., 2005) are sparse and limited in scope. We hypothesize that empirical relationships based on fundamental quantity–intensity–capacity relationships (Beckett & White, 1964), as used in early P models like EPIC, can generalize across diverse agricultural settings and between soils and sediments, thus bolstering our modern P models.

Following Sharpley's long‐term efforts to explain P fate in the environment (Flaten et al., 2024), as well as his collaborative approach to building scientific understanding and consensus around P management (Macrae et al., 2024; Osmond et al., 2024), this study reports a dataset of >600 soils and sediments from seven watersheds in the United States where legacy P threatens water quality. These watersheds cover a variety of pedoclimates, agricultural systems, and P histories while using consistent methodology. Our general goal was to improve the prediction of P lability from soils and sediments that may behave as legacy P sources. We included labile P quantity as P desorbable in 24 h, intensity as the concentration of net zero sorption, and buffer capacity variables, among others, to establish a strong basis for understanding labile P while supporting P model development and hypothesis testing.

**Core Ideas**

- The USDA Legacy P project analyzed >600 soils/sediments across seven participating watersheds.
- Soil labile P stored in top 1 cm at all sites are enough to potentially sustain high P losses for decades.
- Soil P at all sites was vertically stratified, particularly so for labile P, representing a risk for P loss.
- P intensity (equilibrium phosphate concentration at net zero sorption [EPC_0_]) in all soils and sediments was described through a quantity–intensity–capacity relationship.
- Data here can inform the future generations of P transport models needed to address legacy P.

## 2 MATERIALS AND METHODS

### 2.1 Study sites

The seven study sites were selected from active projects within the USDA Conservation Effects Assessment Project (CEAP) Watersheds Assessment Studies Network, constituting the USDA Legacy Phosphorus Project (Table 1). Soil and sediment sampling plans followed a general framework but were site‐specific to test local hypotheses around P mobility and aid model development (Table S1). To exclude soils having recently received fertilizer amendments, the time since the last P application to soils was >1 year (often >3 years), except for one field at the Snake R. site (∼0.5 year).

**TABLE 1:** Watershed and field management information for soils and sediments sampled.

Study watershed (abbreviation), state	MAP (mm)	MAT (°C)	Crop or pasture setting	Historic external P source	Tillage regimes present^a^	USDA soil series	USDA soil orders	Drainage class^b^	Unique soil locations (total soil samples)	Total sediment samples
Lake Champlain (L. Champ.), VT	1010	8.25	Continuous corn; corn–legume–hay	Liquid dairy manure	No till; reduced tillage	Covington (SiC); Vergennes (C)	Alfisols	Poorly drained–moderately well drained	41 (82)	3
Le Sueur river (Le Sueur R.), MN	907	7.57	Soybean–corn	Hog manure, fall injection after soybean	Fall chisel; spring field cultivate	Barbert (SiL); Comfrey (L); Lomax (L); Minneopa (SL); Minnetonka (SiCL); Shorewood (SiCL); Storden (L)	Mollisols, Inceptisols	Very poorly drained–well drained	21 (75)	9
Beasley lake (Beasley L.), MS	1450	18.0	Continuous soybean	Inorganic fertilizer	Conventional; reduced tillage	Alligator (C); Dowling (C); Dundee (L); Forestdale (SiCL); Sharkey (C)	Vertisols; Alfisols; Inceptisols	Very poorly drained–somewhat poorly drained	43 (85)	14
Snake river basin (Snake R.), ID	274	10.1	Alfalfa; barley; corn–sugar beet–barley	Inorganic fertilizer; dairy manure	Conventional	Portneuf (SiL)	Aridisols	Well drained	30 (58)	6
Western Lake Erie basin (W. L. Erie), IN	1030	9.75	Corn–soybean	Poultry litter; inorganic fertilizer	No‐till; conventional	Blount (SiL); Glynwood (SiL); Morley (SiCL); Pewamo (CL)	Alfisols; Mollisols	Very poorly drained–moderately well drained	54 (92)	8
Mahantango creek (Mahan. Ck.), PA	1290	10.2	Continuous pasture; corn–soybean; hay–corn–soybean; continuous corn	Cattle manure; swine manure	No‐till; conventional; reduced tillage	Albrights (SiL); Alvira (SiL); Berks (L); Calvin (L); Leck Kill (SiL); Watson (SiL); Weikert (SiL)	Inceptisol; Ultisols; Alfisols	Somewhat poorly drained–well drained	40 (80)	12
Choptank river, coastal plain (Chop. R.), MD	1270	14.0	Continuous miscanthus	Dairy manure; poultry litter	No‐till; conventional	Corsica (L); Fallsington (SL); Hambrook (L); Woodstown (SL); Zekiah (SiL)	Ultisols	Poorly drained–well drained	45 (90)	10

*Note*: For brevity throughout the paper, sites are only referred to by their abbreviation here. Average climatic data (past 30 years) sourced from Daymet (Oak Ridge National Laboratory). MAP and MAT are mean annual precipitation (mm) and temperature (°C); typical soil texture abbreviations are clay (C), silt (Si), sand (S), and loam (L).

^a^ Simplifiers “conventional” (e.g., multiple operations of plowing, disking) and “reduced” (e.g., fewer passes and with less‐intensive plowing) are used here, but are relative to the region and cropping system. Table S1 provides some further detail.

^b^ Drainage classes are sourced from SSURGO and may overlook the presence of subsurface drainage, particularly at the Le Sueur and W. L. Erie sites where drainage is poor.

### 2.2 Soil and sediment sampling

All sampling occurred during the spring of 2022 and followed a standard protocol for collecting and processing samples. At each sampling location, soil samples were collected at depths of 0–5 cm and 5–15 cm over five or more cores and homogenized to a composite sample. Depending on site‐specific hypotheses, deeper soil layers and bank sediments were sampled; 16 bank sediments at W. L. Erie were treated as soils based on site context. Ditch and stream sediments near the soil sampling sites were collected from approximately 0–2 to 0–5 cm of substrate across more than five locations within the main channel in a 5–10 m stretch, homogenized, and wet‐sieved to <2 mm in the field. Sediment samples targeted zones of interaction with overlying water and avoided deeper, likely anoxic, sediments. Generally, soils were sieved to <2 mm; however, some difficult (i.e., high clay content) soils under field‐moist conditions (for the frozen pretreatment, below) required sieving to <4 mm. We treated all samples as equivalent at the <2 mm threshold since the high‐clay samples had minimal, if any, particles >2 mm.

Immediately after collection, all samples were stored on ice and in the dark. Each soil and sediment sample (∼0.5 kg) was split into two subsamples based on pretreatment: air‐dried and frozen. The frozen pretreatment (−20°C) was chosen to minimize the effects of drying and storage on sensitive analyses (e.g., aging of redox‐sensitive metal oxides; Z. P. Simpson et al., 2019). Frozen samples were thawed immediately prior to analysis. Air‐drying was used for practicality in other analyses as well as to match historical data. An exception to the frozen pretreatment was 30 soils (Snake R.) that were excluded based on site‐specific grounds.

### 2.3 Soil and sediment analyses

All analyses except for particle size distribution were performed in a common laboratory using the methodology outlined in Table 2. Laboratory quality assurance/quality control for methods mentioned here are detailed in the Supporting Information. All extractions were performed at standard lab temperature of 20 ± 1°C. Each analysis batch included a subsample of random duplicates and quality control checks; three reference soils from the North American Proficiency Testing program were also included in each batch to ensure accuracy and replicability. When describing an extractable element concentration, we use subscripts denoted in Table 2 (e.g., Olsen‐extractable P is P_Ols_). Analyte mass concentrations are given as mass per dry weight throughout.

**TABLE 2:** Summary of analyses for soils and sediments.

Analysis	Sample preparation	Method summary	Comments	Reference
pH	Air‐dried	1:1 or 1:2 in deionized water, 30 min	Ratio dependent on clay content of slurry	(R. O. Miller & Kissel, 2010)
Particle size distribution	Air‐dried	Laser diffraction, triplicate samples averaged	Clay‐silt distinction at 6 µm	(Faé et al., 2019; B. A. Miller & Schaetzl, 2012)
Total C/N, total organic and inorganic C (TC, TN, TOC, TIC)	Air‐dried	Combustion/pyrolysis	Total C and N measured at 900°C; total organic C measured at 600°C	(ISO, 2024)
Olsen P extraction (Ols)	Air‐dried	1:20, 0.5 M NaHCO_3_ at pH 8.5, 30 min, followed by ICP‐OES	Filtered through Whatman 1 filter paper	(Olsen et al., 1954)
Mehlich‐3 extraction (M3)	Air‐dried	1:10, Mehlich‐3 extraction solution (pH 2.5), 5 min, followed by ICP‐OES	Filtered through Whatman 1 filter paper	(Mehlich, 1984)
EPA 3050B digestion for total elements (tot)	Air‐dried	1:3, conc. HNO_3_ and HCl with 30% H_2_O_2_ followed by ICP‐OES	Performed on automated digestion block	(EPA, 2017)
Labile P: Desorption by anion exchange membrane (AEM)	Air‐dried	2 g sample plus 6.5 cm^2^ exchange membrane (Cl^−^ form; SnowPure Water Technologies Excellion I‐200 anion membrane) in 50 mL deionized water, 24 h, followed by ICP‐OES	AEMs pre‐saturated >24 h before use; Membrane P eluted by 1 h shaking in 1 M NH_4_Cl; the AEM size targeted sorption capacity of ∼7000 mg P kg^−1^ soil	(Cooperband & Logan, 1994; Sharpley et al., 1984; Sibbesen, 1977)
Acid ammonium oxalate extraction (ox)	Frozen	1:40, 0.2 M (NH_4_)_2_C_2_O_4_ buffer_,_ pH 3.0, 2 h, followed by ICP‐OES	Conducted in the dark to avoid potential reagent photolysis	(McKeague & Day, 1966)
Bicarbonate‐dithionite extraction (BD)	Frozen	1:20, 0.1 M NaHCO_3_ + NaS_2_O_4_ (pH > 7) for 2 h, followed by ICP‐OES	Extract acidified to prohibit metal precipitation followed by 24 h of passive aeration; Al_BD_ were <0.8% Al_tot_	(Jan et al., 2015)
Equilibrium phosphate concentration at net zero sorption (EPC_0_)	Frozen	1:20, varying PO_4_ concentrations in CaCl_2_ + NaCl background, 16 h, followed by ICP‐MS	Background solutions were 0.5 mM NaCl and 1.5 mM CaCl_2_ for alkaline soils, and 0.5 mM NaCl and CaCl_2_ for all others.	(Z. P. Simpson et al., 2021; Taylor & Kunishi, 1971)
Single point sorption indices (*S*_max_; BWI)	Frozen	1:20, 150 mg P L^−1^ in EPC_0_ background (above), 16 h, followed by ICP‐OES	Samples were agitated for 18 h	(Bache & Williams, 1971; Bolster et al., 2020)

*Note*: Ratios indicate soil/sediment to solution (mass basis); mentions of sample masses are given on a dry weight basis. Times are for extraction/equilibration.

Abbreviations: BWI, Bache–Williams index; ICP, inductively coupled plasma; MS, mass spectrometry; OES, optical emission spectroscopy.

We determined degree of P saturation (DPS) by two different methods: (1) the original molar ratio of P_ox_ to the sum of Al_ox_ and Fe_ox_ (DPS_ox_); (2) the same ratio but via the Mehlich‐3 extraction (DPS_M3_; Kleinman & Sharpley, 2002). Following Kleinman (2017), no empirical correction was applied to the denominator in either DPS. Additionally, we calculated the ratio of total OC to the sum of Al_ox_ and Fe_ox_, OC:(Al + Fe)_ox_ (mol mol^−1^).

Labile P was measured as desorption in presence of a P sink via anion exchange membrane (P_AEM_), similar to anion exchange resin methods (protocol in Supporting Information). The 24‐h desorption period was chosen to reflect the definition of labile P in Jones et al. (1984), which includes readily exchangeable P on the timescale of several hours to a day but shorter than the days to weeks timescale implied for the “active” pool.

The equilibrium phosphate concentration at net zero sorption (EPC_0_) was determined for all soils and sediments. Details on methodology and statistical estimates of EPC_0_ are in the Supporting Information (see also Tables S2 and S3; Figure S1). The net P sorption was modeled as a function of initial P concentration (Z. P. Simpson et al., 2019), where the *x*‐intercept is the EPC_0_; samples from the same site were combined in a multilevel model to improve the accuracy in EPC_0_ via partial pooling (McElreath, 2020).

Single point sorption indices estimate the maximum sorption capacity parameter in the Langmuir isotherm; the mass of P sorbed is the sorption maximum (*S*_max_). The methodology as given by Bolster et al. (2020) was followed but with two changes. We used the same background matrix as for EPC_0_ and chose a 150 mg P L^−1^ concentration, rather than the 200–300 mg P L^−1^ recommended by Bolster et al. (2020), to avoid potential artifacts of excess P precipitation for alkaline samples (Leytem & Westermann, 2003). We used these same data to calculate the Bache–Williams Index (BWI; Bache & Williams, 1971), which is a P buffering capacity metric: BWI = *S*_max_/log(*c*_equil_), where *c*_equil_ is the final solution equilibrium P concentration; units are mg P hg^−1^ per log(µM).

### 2.4 Data and analysis

Additional soil information was queried from SSURGO (Soil Survey Geographic Database; NRCS Soil Survey Staff, 2024) and the clay mineralogy dataset of Ito and Wagai (2017). We focus here primarily on general relationships in the data but did test, where appropriate, for the effects of land use, tillage regime, and time since last P application (recorded at site level as practical). However, as our sampling design did not necessarily stratify across these factors, our inferences here are limited. We consider the time since last P application (usually >1 year) to have negligible effect here (the spike in labile P following P addition wanes after days to weeks; Jones et al., 1984; Sharpley, 1982), which is discussed further in Supporting Information, and is therefore not considered further.

As a way to contextualize the labile P data with empirical runoff data, we reexpress these mass concentrations as stocks: P_AEM_ stock (kg ha^−1^) = P_AEM_ (mg kg^−1^) × bulk density (g cm^−3^) × depth (cm) × 0.1, where 0.1 is a conversion factor. We made three assumptions in this calculation. First, we used a depth of 0–1 cm by assuming that P_AEM_ measured in the topsoil (0–5 cm) or surface sediment (∼0–3 cm) is equivalent to that of the 0‐ to 1‐cm depth; this is conservative if vertical P stratification exists, as P concentration in 0–1 cm is likely greater than that below. Second, we assumed a depth of 0–1 cm to reflect a conservative depth of interaction with runoff or the water column, even though it is likely greater for both soils (Sharpley, 1985a) and sediments (Boano et al., 2014). Third, to simplify calculations, we assumed a bulk density of 1.2 g cm^−3^. Bulk density possibly varies from approximately 0.9 to 1.5 g cm^−3^ for these samples, but this variation has very small leverage on the P_AEM_ stock compared to P_AEM_ concentrations.

Analyses were performed in the R statistical language, v. 4.3.2 (R Core Team, 2023). We report Spearman's test for correlation (*ρ*); for reference, the critical values (95% level) of *ρ* are |*ρ*| ≥ 0.373 and |*ρ*| ≥ 0.197 for sample sizes of 28 and 100, respectively. For pairwise contrasts and related model‐based comparisons, we used the “emmeans” package (Lenth, 2023). To judge between competing models for our varied analyses, we used visual diagnostics, residual behavior, and out‐of‐sample prediction error statistics such as Akaike information criterion (AIC) or leave‐one‐out information criterion, following best practice (Burnham & Anderson, 2004; Vehtari et al., 2017).

For quantifying contrasts between soils and sediments, we fit generalized linear models that included site, sample type, and sand concentration (as a control for texture; dropped for clay concentration contrasts). To keep these contrasts simple, we compared models with the Gaussian (pH only) or the gamma (using log link) families, which helped in cases of heteroskedasticity and skewness.

In testing for stratification of soil P, we used soil samples from the 0‐ to 5‐ and 5‐ to 15‐cm depths and calculated their ratio in P concentrations for P_AEM_, P_M3_, P_Ols_, and P_tot_. As ratios are naturally skewed, we fitted models using the gamma family with log link; we included site, tillage intensity, and land use as predictors, although some models suggested variation in stratification ratios were primarily due to site.

We built models for EPC_0_ as a function of P_AEM_ and related buffer variables using generalized additive models (GAMs; Wood, 2017), in line with our quantity–intensity–capacity hypothesis. As EPC_0_ is constrained positive and featured large skewness, we modeled EPC_0_ as gamma distributed (with log link). We tested a variety of models ranging in complexity but centered around the hypothesis that intensity is ultimately a function of quantity and buffer capacity. We represented nonlinear relationships with regularized smooth spline functions. To test if a general model outcompetes a site‐specific model, we also fitted models featuring by‐site smooth functions for the EPC_0_‐P_AEM_ relationship (Pedersen et al., 2019).

All data and R code are published through Ag Data Commons (https://doi.org/10.15482/USDA.ADC/25892602.v1).

## 3 RESULTS AND DISCUSSION

### 3.1 Soil and sediment characteristics

**FIGURE 1:**
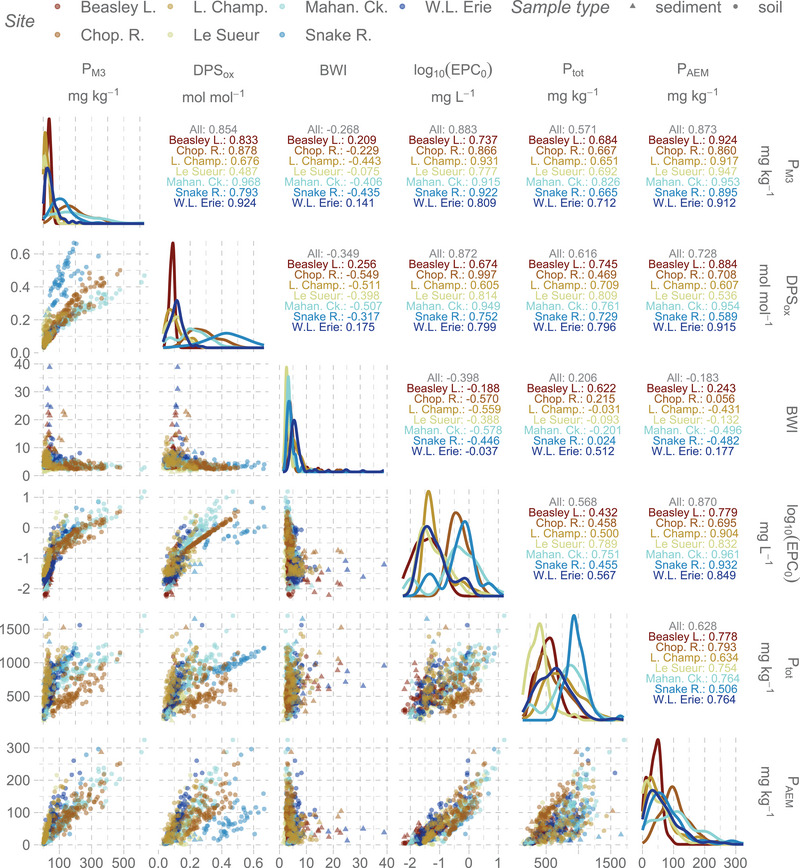
(A) correlation matrix for select P‐related variables for soils and sediments across the seven study sites. Values in upper triangle are Spearman's rho (*ρ*) correlations (across both soils and sediments). Diagonal plots are the marginal distributions for each variable. Equilibrium phosphate concentration at net zero sorption (EPC_0_) is given as its log_10_ for visibility. Bache–Williams index (BWI) has units of mg P 100 g^−1^ per log(µmol P L^−1^). Indices for statistical significance are omitted here for clarity, but as a reference, |*ρ*| ≥ 0.197 and |*ρ*| ≥ 0.338 are the critical values at the 95% confidence level for sample sizes of 100 (max per site) and 34 (minimum sample size here, corresponding to EPC_0_ at Snake River); most tests here are for sample sizes of 64–100. AEM, anion exchange membrane; DPS, degree of phosphorus saturation.

The soils and sediments sampled represent six soil orders and 31 distinct soil series with varying mineralogy, drainage classes, slopes, and textures, resulting in large variability across physicochemical and sorption properties (Table 3; see also Supporting Information, Figures S2–S5). Labile P concentrations, as measured with a 24 h desorption (P_AEM_), were typically (interquartile range) 34–100 mg P kg^−1^ but varied as widely as ∼200–300 mg P kg^−1^ depending on the site (Figure 1); for comparison, previous large‐scale studies measured labile P as large as 43–56 mg P kg^−1^ (depending on weathering status; Sharpley et al., 1984), 26–90 mg P kg^−1^ (depending on region in the United States; Wolf et al., 1985), and up to 750 mg P kg^−1^ in manured soils (Sharpley et al., 2004). Labile P was highly correlated to EPC_0_ among sites but inconsistently correlated to BWI (Figure S4).

**TABLE 3:** Major soil (all depths; *n* = 562) and sediment (*n* = 62) characteristics, total element concentrations, extractable element concentrations, and sorption properties for seven study watersheds across the United States.

		Soil			Sediment		
Variable	Unit	Median (mean)	Min–max	IQR	Median (mean)	Min–max	IQR
pH	S.U.	6.75 (6.81)	4.83–8.73	6.26–7.3	6.87 (6.96)	5.19–8.7	6.06–7.78
Total organic C	g kg^−1^	15.5 (18.8)	3.15–84.6	11.6–22.2	15.8 (21.6)	2.13–95.7	7.85–27.3
Total inorganic C	g kg^−1^	0 (2.82)	0–41.6	0–2.59	0 (5.26)	0–36.1	0–10.2
Clay	g kg^−1^	360 (362)	88–890	225–464	168 (236)	32.2–564	121–311
Sand	g kg^−1^	233 (260)	0–827	166–324	446 (452)	84.2–917	295–586
DPS_ox_	mol mol^−1^	0.142 (0.189)	0.031–0.667	0.091–0.261	0.110 (0.125)	0.047–0.413	0.084–0.14
DPS_M3_	mol mol^−1^	0.075 (0.264)	0.003–0.674	0.035–0.199	0.056 (0.096)	0.013–0.452	0.035–0.106
OC:(Al + Fe)_ox_	mol mol^−1^	19.9 (21.4)	2.96–79.0	12.8–28.4	17.3 (25.7)	5.63–161	11.7–30.0
Total P	mg kg^−1^	650 (680)	168–1700	467–878	543 (599)	142–1650	332–730
Total Fe	mg kg^−1^	20.6 (20.4)	1.58–50.5	14.9–24.9	18.8 (18.6)	3.20–41.4	10.7–24.5
Total Al	mg kg^−1^	16.3 (17.5)	4.15–51.8	13.0–20.5	11.4 (14.0)	2.64–36.8	6.94–20.3
Total Ca	mg kg^−1^	3.06 (6.38)	0.479–73.6	1.99–4.64	3.31 (17.7)	0.397–130.	1.50–25.1
Olsen P	mg kg^−1^	31.3 (34.7)	6.2–136	20.8–43.1	20.5 (29.8)	4.16–115	8.76–47.8
Mehlich‐3 P	mg kg^−1^	58.9 (102)	2.6–628	31–146	29.1 (44)	4–360	18.1–39.9
Mehlich‐3 Al	mg kg^−1^	638 (618)	2.6–1190	529–756	419 (404)	0.18–971	190–669
Mehlich‐3 Fe	mg kg^−1^	224 (239)	20.5–649	160–318	406 (375)	39.1–683	229–525
Ammonium oxalate P	mg kg^−1^	359 (384)	80.5–1270	255–497	261 (310)	48.1–1240	134–427
Ammonium oxalate Fe	mg kg^−1^	2250 (2680)	330–10200	1150–3890	2360 (3230)	431–10300	1380–4940
Ammonium oxalate Al	mg kg^−1^	874 (978)	205–3170	680–1220	585 (686)	105–2910	300–919
Bicarbonate dithionite P	mg kg^−1^	44.6 (61.5)	3.36–355	20.1–89.8	52.9 (62.7)	9.82–311	24.4–83.6
Bicarbonate dithionite Fe	mg kg^−1^	2480 (2570)	326–7800	1330–3510	1670 (2190)	281–5970	928–3130
Bicarbonate dithionite Fe:P	mol mol^−1^	27.7 (58.6)	1.23–591	10.6–74.6	13.6 (38.3)	1.65–330	10.5–27.5
24 h P desorption via AEM	mg kg^−1^	65 (77.8)	4–325	36.4–108	32.6 (43.7)	2.9–288	16.6–53.8
*S*_max_	mg kg^−1^	331 (368)	159–1740	262–422	541 (732)	111–2320	351–1000
Bache–Williams index	(mg P 100 g^−1^) per log(µmol P L^−1^)	3.99 (4.48)	1.9–22.6	3.16–5.14	6.71 (9.58)	1.35–38.8	4.26–12.6
EPC_0_	mg L^−1^	0.158 (0.521)	0.0057–15.6	0.0466–0.53	0.0344 (0.0758)	0.00778–1.43	0.0192–0.0631

*Note*: IQR is interquartile range; EPC_0_ is the equilibrium phosphate concentration at net zero sorption; *S*_max_ is the sorption maximum in a Langmuir isotherm; DPS is the degree of P saturation (ratio of P to Fe + Al) in either Mehlich‐3 or ammonium oxalate extracts; AEM is anion exchange membrane.

The BWI, a measure of the P buffer capacity of soils and sediments (Bache & Williams, 1971), had similar medians across sites (3.2–5.2) but maximum BWI varied from 6.7 (Le Sueur) to 39 (W. Lake Erie). Among sites, the BWI was negatively correlated with pH except at sites with more alkaline soils (Snake R., W. Lake Erie); positively correlated with Fe_ox_ + Al_ox_ (except at Snake R.); and variably correlated (negative, none, or positive) with OC:(Al + Fe)_ox_, DPS_ox_, Fe_BD_:P_BD_, and clay content. This reinforces clay minerals and hydrous metal oxides as major sorption sites for P (via ligand exchange) in soils and sediments, particularly for more weathered settings but, in more alkaline settings, precipitation (e.g., Ca‐based) reactions also contribute to P buffering (Hartikainen et al., 2010; Ige et al., 2005; Sharpley, 1983; Z. P. Simpson et al., 2022).

Possible effects of land management on P properties were varied and highly site‐specific (see Supporting Information; Figures S6–S9), which will require further study at the site level. Time since last P fertilizer application had no consistent effect on core P variables, particularly the relationship between EPC_0_ and labile P (Figure S6), which suggests that labile P was near an equilibrium regarding recent P inputs. This equilibration behavior is expected for timescales >180 days (van der Zee & van Riemsdijk, 1988) and is consistent with the ∼60‐ to 90‐day window for elevated P losses following P application observed in edge‐of‐field runoff (McDowell et al., 2003; Osterholz et al., 2024).

**FIGURE 2:**
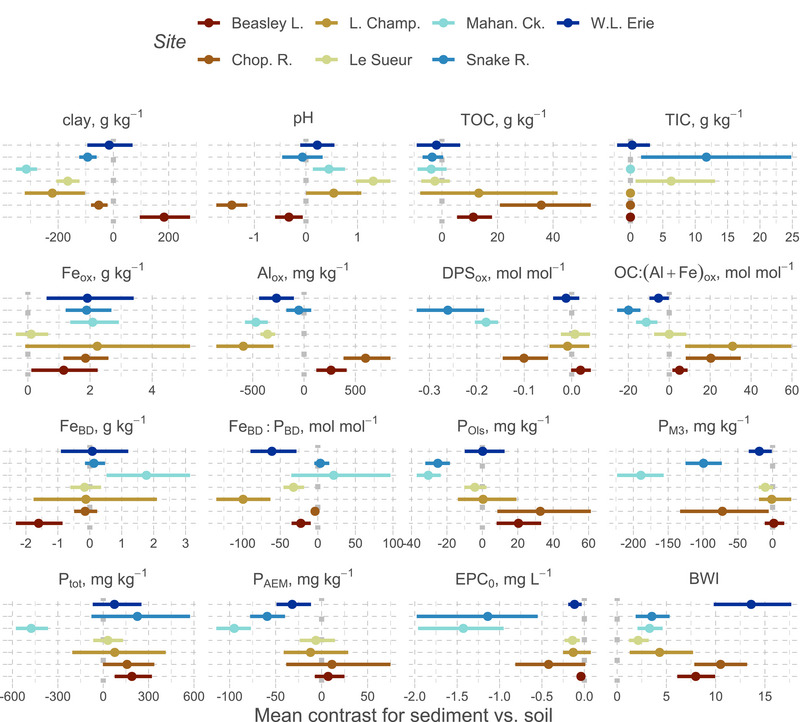
Selected contrasts of soil and sediment means for variables at each site. The *x*‐axis represents the sediment value minus the soil value. Points (estimated marginal means) to the left of the vertical dashed line (<0) indicate soils having a greater mean value compared to sediments. Except for clay concentration, contrasts are based on models accounting for sand concentration. Error bars represent a 95% confidence interval. BWI is Bache–Williams index. AEM, anion exchange membrane; DPS, degree of phosphorus saturation; EPC_0_, equilibrium phosphate concentration at net zero sorption; OC, organic carbon; TIC, total inorganic carbon; TOC, total organic carbon.

The combined analysis of both soils and sediments addresses the objective of determining generalized relationships; however, these samples differ substantially (Figure 2). Generally, sediments were coarser in texture than soils (likely due to particle sorting during transport) except at Beasley L. Depending on sand concentration, sediments were more sorptive than soils (BWI differences of 2.3–13.5) and likewise sediment EPC_0_ were often less, though this may be driven by the more extreme soil samples for some sites (e.g., Mahan. Ck. had four soils with EPC_0_ >5 mg L^−1^). More consistent contact and therefore exchange with flowing solution for sediments likely drives this contrast in EPC_0_ (Z. P. Simpson et al., 2021). Agudelo et al. (2011) observed a similar pattern for EPC_0_ as well as for labile P when comparing sediments to upland soils. In our study, labile P was greater in soils than in sediments at Mahan. Ck., Snake R., and W. L. Erie (differences of 32–95 mg kg^−1^). However, labile P concentrations in sediments were still large, with medians varying from 9.6 (Mahan. Ck.) to 91 (Chop. R.) mg kg^−1^, representing a significant source of P, which may slowly release to overlying waters. Contrasts between soils and sediments in properties including pH, Fe, and P concentrations were highly site‐specific, reflecting the context of the landscapes sampled.

Soil and sediment properties, as they pertained to P concentrations and sorption properties, correlated well (Figure 1; Figures S3 and S4). Typically, greater P_M3_ or P_Ols_ coincided with greater P_AEM_, EPC_0_, and DPS_ox_, weakly but still positively correlated with P_tot_, and negatively correlated to BWI. Though most of these variables were right skewed, EPC_0_ was severely so. While P_tot_ remains important as it accounts for the full amount of P in these systems, the data here clearly suggest that the “availability” of this P varies considerably across contexts. For example, depending on the site, the percentage of P_tot_ present in the various extractions (medians) were 6.3%–19.2% (P_AEM_), 2.7%–33% (P_M3_), 3.1%–7.4% (P_Ols_), 45%–70% (P_ox_), and 2.5%–16% (P_BD_); while we lack data to test this more strictly, there was only little evidence that land use was predictive of these proportions (Figure S5). However, the P history of these samples is evident: for comparison, in soils without anthropogenic P inputs in the review by Cross and Schlesinger (1995), labile P comprised at most 6% of total P. Phosphorus storage in these extractable pools varied considerably across and within sites not only due to management history, but due to, for example, potential for mineral precipitation/dissolution (Ippolito et al., 2019), geology or parent material (McDowell, 2015; Z. P. Simpson et al., 2022), and redox regime (Couic et al., 2022; Sandström et al., 2021; Smith et al., 2021). These factors and more deserve investigation at the site level.

### 3.2 Vertical P stratification in soils

**FIGURE 3:**
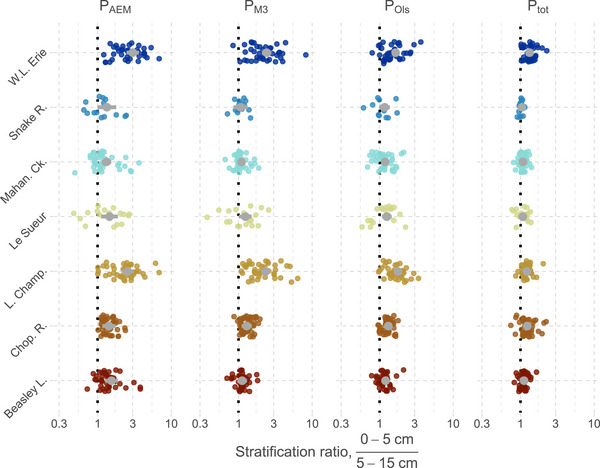
Vertical stratification ratios for labile P (P_AEM_), Mehlich‐3, Olsen, and total P across the seven sites. The ratio is that of the 0–5 cm layer to the 5–15 cm layer at the same sampling location; a value of 1 (dotted line) suggests little stratification of the soil property for this depth range. Colored points are individual soil locations per site. Gray points are the model‐based means (lines are 95% confidence intervals) conditional on land use being agricultural while marginalizing over any effect of tillage intensity (which was small). AEM, anion exchange membrane.

Vertical P stratification in surface soils is a common feature in agricultural landscapes with a history of P application and minimal soil mixing (Baker et al., 2017; Sharpley, 2003), and P‐stratified soils may contribute more P to runoff compared to less P‐stratified soils (Kleinman et al., 2022). Soils collected here involved two standard depths across sites (0–5 and 5–15 cm), whose ratio depicts stratification in several P pools (Figure 3). Stratification in soil P was largely influenced by site‐specific variation rather than apparent tillage intensity or land use; however, follow‐up studies at the site‐level may explain the role of these factors. For P_M3_ at five sites, topsoils (0–5 cm) were enriched relative to subsoils (5–15 cm) by factors of 1.15 (95% confidence interval [CI]: 1.00, 1.31; Beasley L.) to 2.44 (95% confidence interval [CI]: 2.14, 2.79; W. Lake Erie) but not for Snake R. or Mahan. Ck. The stratification ratio was also negligible at Snake R. for P_Ols_ and P_tot_, likely due to regular tillage and root crop harvest; however, for the other six sites, stratification in P_Ols_ ranged from 1.14 (95% confidence interval [CI]: 1.00, 1.30; Mahan. Ck.) to 1.71 (95% confidence interval [CI]: 1.55, 1.88; L. Champ.), while stratification in P_tot_ ranged from 1.11 (95% confidence interval [CI]: 1.02, 1.21; Le Sueur) to 1.37 (95% confidence interval [CI]: 1.29, 1.45; W. L. Erie).

In contrast, labile P was much more stratified at all sites compared to other P pools, ranging from 1.34 (95% confidence interval [CI]: 1.07, 1.69; Mahan. Ck.) to 3.05 (95% confidence interval [CI]: 2.57, 3.64; W. L. Erie) times greater concentrations of P_AEM_ in topsoils. Stratification ratios for P_M3_ tended to be more variable compared to the other P pools, suggesting that stratification in P_M3_ can be highly sensitive for some sites (L. Champ.) yet surprisingly mute for others with clear stratification by P_AEM_ or P_tot_ (e.g., Beasley L. and Mahan. Ck.). The greater degree of vertical stratification in labile P than in other P pools may be related to the concentrations of P relative to sorption sites in surface soils (Figure S10). While BWI, Fe_ox_, Al_ox_, and Fe_BD_ were relatively unstratified between soil layers, stratification in soil P was well‐correlated to stratification in DPS_ox_ and Fe_BD_:P_BD_. For some sites, labile P stratification also correlated with that of OC:(Al + Fe)_ox_ (excluding Le Sueur and Mahan. Ck.), which may indicate that changes in organic matter with depth could alter sorption site availability (Achat et al., 2016).

### 3.3 Labile P quantity in soils and sediments

Phosphorus that is “labile,” defined here as soil or sediment surface P readily able to exchange with solution P (Pierzynski et al., 2005), is a core component in predicting dissolved P losses from soils and sediments to surface waters (Sharpley, 1985b; Sharpley et al., 1984, 2004). Our dataset provides a measure of the quantity of labile P, which is the mass of P that can release into solution in 24 h under optimal conditions (P_AEM_) following the definition used in EPIC, SWAT, and related models (Jones et al., 1984; Vadas & White, 2010).

**FIGURE 4:**
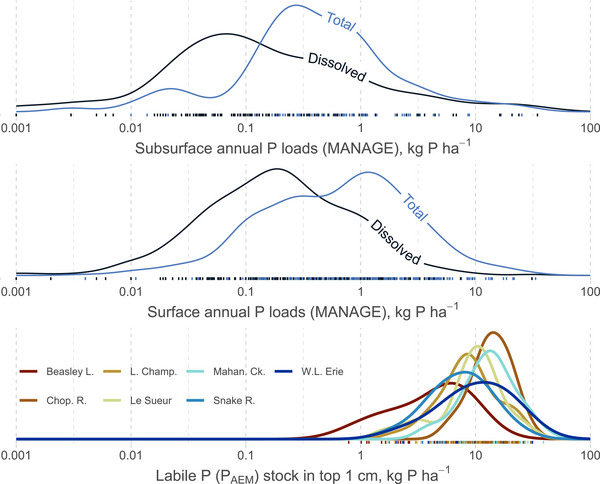
Probability densities for surface soil stocks of labile P (P_AEM_) across sites compared to typical annual P losses from fields in the MANAGE dataset (Harmel et al., 2017). Annual P losses (medians per each distinct site in MANAGE) are separated by subsurface/tile (top) and surface (middle) pathways and by total P and dissolved P forms. The stocks of P_AEM_ (bottom) assume the top 1 cm of soil and a bulk density of 1.2 g cm^−3^. See Figure S11 for comparison to sediment labile P stocks. AEM, anion exchange membrane.

One reason labile P, and its connection to legacy P, is critical is that it is a strong determinant of P loss in runoff (Sharpley, 1983): P loss increases with both greater hydrologic transport and with greater source of labile P (Jones et al., 1984; Kleinman et al., 2006; Sharpley, 1985a). To put the source, as P_AEM_, into more context, we calculated the P_AEM_ stock for agricultural topsoils (excluding “reference” soils) across all sites and compared these to typical (median) annual P losses at the field scale via the MANAGE dataset (Harmel et al., 2017, 2022). Clearly, each of the seven sites here contain soils that can potentially sustain P loads rivaling the largest dissolved P losses (>75th percentile of 0.5 kg P ha^−1^) reported across MANAGE for decades or longer (Figure 4). Similarly, the majority of surface sediments stored more than 1 kg P ha^−1^ in labile P (Figure S11). Losses at this scale can be harmful: for one comparison, the Chesapeake Bay total maximum daily load for annual total P loss across the basin is 0.34 kg P ha^−1^ (U.S. EPA, 2010). Field sites similar to those reported here (including some in MANAGE) often do not exceed annual P losses of 10 kg P ha^−1^, despite likely having more than adequate labile P stocks, since P loss in these contexts is often transport limited (cf. supply‐limited; Basu et al., 2011; Buda et al., 2009; Williams et al., 2022). Stated differently, P losses in these catchments may persist for decades unless the supply driving P fluxes—labile P—is diminished. Thus, it is of great concern that many catchments globally are still accumulating more P than they are exporting (Boardman et al., 2019; McCrackin et al., 2018; Stackpoole et al., 2019).

Labile P measured as P_AEM_ is not common in the literature, particularly for a similar number of samples as here. Prior work on labile P relationships has focused on water‐extractable P (WEP), CaCl_2_‐extractable P, and other intensity‐based extractions as target measures of labile P, often finding that these generally correlate with soil test P or with DPS (Blombäck et al., 2021; Koopmans et al., 2002; McDowell, Sharpley, et al., 2001). These different measurements of soil and sediment P, more intensive than extensive, can correlate to labile P quantity, but they do not predict its full extent. We argue that the P_AEM_ measurement, as an extensive variable, can give a more correct estimate of the quantity of P stored on soil/sediment surfaces that can readily release to solution (Maertens et al., 2004; Sharpley et al., 1984). This is achieved through including a P sink in the extraction, such that the process of P desorption continues throughout the period. In contrast to variables like soil test P, WEP, and CaCl_2_‐P, P_AEM_ is not overly sensitive to methodological parameters like mass‐to‐solution ratio. Future work here could improve methods for initializing labile P in transport models, which often rely on general conversions of soil test P to labile P (Vadas & White, 2010).

### 3.4 Revisiting quantity–intensity–capacity relationships

The models we use to understand the impacts of legacy P rely heavily on quantity–intensity relationships (Pierzynski et al., 2005; Radcliffe & Cabrera, 2006). While the labile P pool is the *quantity* of readily‐exchangeable P on the solid surface, the *intensity* is the solution P concentration the labile P pool supports as mediated by the *buffer capacity* (Beckett & White, 1964). The intensity variable we measured here is the EPC_0_, which is the equilibrium solution phosphate potential governed by the soil or sediment surface (White & Beckett, 1964), and this concentration relative to solution P dictates the direction of sorption processes in the environment (Z. P. Simpson et al., 2021). Unlike other intensity‐based variables discussed above (WEP and CaCl_2_‐P), EPC_0_ is not sensitive to the mass‐to‐solution ratio, making it a more ideal estimate of intensity. To our knowledge, this dataset is the largest pairing of both EPC_0_ and labile P.

**FIGURE 5:**
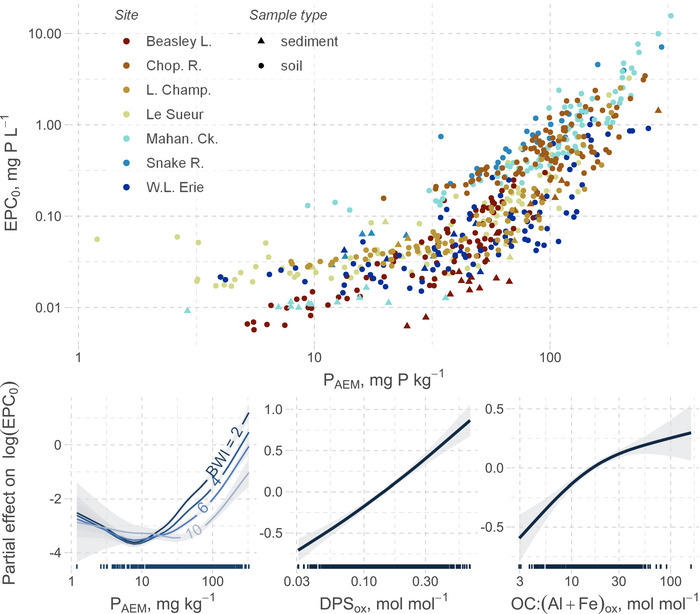
Soil and sediment equilibrium phosphate concentration at net zero sorption (EPC_0_) as a function (top) of labile P (P_AEM_) forms a coherent quantity–intensity relationship (where EPC_0_ is the intensity variable). This relationship is further moderated by buffer capacity, where EPC_0_ modelled with a generalized additive model (GAM) (bottom; additive model terms arranged in decreasing order of influence) as a function of labile P interacts with Bache–Williams index (BWI) while increasing amounts of degree of P saturation or organic carbon relative to potential sorption sites (DPS_ox_ and OC:(Al + Fe)_ox_, respectively) can increase EPC_0_. Plots on the bottom show the (additive) partial effects within the GAM which are given on the log‐scale; shaded areas are standard errors and rug plots on *x*‐axis are marginal distributions of the predictor. AEM, anion exchange membrane.

In a modeling exercise, we used a GAM to show a remarkably consistent relationship between EPC_0_ and labile P, where EPC_0_ increases with greater quantity of P_AEM_, particularly for P_AEM_ > 10 mg kg^−1^ (Figure 5; Table S4). The GAM further identifies, as predicted (Bache & Williams, 1971; Beckett & White, 1964; Holford, 1997), that the buffer capacity as BWI has a strong influence on the quantity–intensity relationship, where greater BWI for a given quantity of labile P decreases EPC_0_. Put another way, for a given intensity of EPC_0_, a greater buffer capacity implies a larger pool of labile P. Across the dataset, we also find a positive effect of both DPS_ox_ and OC:(Al + Fe)_ox_ on EPC_0_. Both of these variables moderate buffer capacity, where higher values likely indicate greater occupation of sorption sites by either P or OC, respectively. While the effect of DPS_ox_ was consistently near log‐linear, the effect of OC:(Al + Fe)_ox_ appeared to diminish after ∼50 mol mol^−1^.

The tight relationship between EPC_0_, labile P, and buffer capacity variables across diverse soils and sediments is encouraging and highlights the ubiquitous role of quantity–intensity–capacity relationships for environmental P management. Not only does the model represent the data well (92.4% deviance explained), it also greatly outperforms models relying on site‐specific relationships for EPC_0_ (ΔAIC = −235), thus supporting our hypothesis that the quantity–intensity–capacity relationship can generalize well. Even for sites with more alkaline pH soils/sediments (Snake River, Le Sueur, and W. Lake Erie), the model is accurate despite the likely increasing importance of Ca‐based precipitation mechanisms for P availability—previously, studies such as Sharpley (1983) segregated empirical relationships by soil pH. Further, alternative GAMs with smooths dependent on soil versus sediment did not outcompete the final model, suggesting that once conditioned on the core P quantity and buffer capacity predictors, both soil and sediment P intensities (EPC_0_) can be predicted. Following P_AEM_ and BWI, the DPS_ox_ and, to a lesser degree, OC:(Al + Fe)_ox_, had a considerable influence on EPC_0_, likely since the saturation of sorption sites diminishes the material's ability to retain more P (Achat et al., 2016; Kleinman, 2017). Similar to the effect of OC:(Al + Fe)_ox_, in a survey of forest soils across drainage classes, Lyons et al. (1998) found a strong dependence of EPC_0_ on organic matter and Al_ox_ + Fe_ox_. These saturation variables are a useful addition since they may be more responsive to management than BWI. Alternative predictors for EPC_0_ such as pH, clay, total organic C, and Fe_BD_:P_BD_ were either unnecessary (no improvement in AIC) or confounded the effects of the other buffer‐related variables. This suggests that differences in properties such as texture, redox environment, and pH are accounted for when predicting EPC_0_ as a function of labile P and buffer capacity.

Given the central role of P intensity for environmental P losses (McDowell & Condron, 2004; Pierzynski et al., 2005), a generalizable quantity–intensity–capacity relationship can bolster the modeling and managing of inorganic P for both soils and sediments (Koopmans et al., 2002; Radcliffe & Cabrera, 2006). Outside of erosion, the transport of P to runoff or the overlying stream, as dissolved P, depends on P in solution, which then depends on the gradient in P intensity (ratio of EPC_0_ to solution P). Dissolved P loss is important to many ecological contexts (Carpenter et al., 1998; Dodds & Smith, 2016), but it is also a major proportion of total P losses: using the MANAGE dataset again (Figure 4), dissolved P loads are typically 20%–70% of total P load depending on the site. Current P transport models help predict dissolved P loads primarily as a function of labile P pools (Das et al., 2019), but future work should expand the focus from *quantity* to also include *intensity*. Robust measurements of P intensity have not always been available in past work, so our dataset can help fill this research gap.

While managing the quantity and intensity of soil and sediment P in our systems is critical, so too is the approach to buffer capacity. Buffer capacity affects agronomic P management, where a strong soil P buffer can mute responses in crop P availability following inputs (R. J. Simpson et al., 2011). However, buffer capacity also affects environmental P management, where strong buffers and a buildup of labile P can sustain high P concentrations (intensity) in aquatic (Haggard et al., 2004; Jarvie et al., 2005; Z. P. Simpson et al., 2021) and terrestrial (Kleinman et al., 2011; Sharpley et al., 2013) environments. Indeed, it is due to the ability of soils and sediments to buffer P inputs that responses to watershed P management are so often lackluster and lagged, leading to legacy P challenges decades after conservation practices are implemented (Meals et al., 2010; Sharpley et al., 2013).

## 4 CONCLUSIONS

Legacy P is a grand challenge for watershed management (Sharpley et al., 2013) and it connects to eutrophication through the labile P pool. This labile P quantity, which can exchange quickly with solution P until the EPC_0_ (intensity) is reached, underpins the conceptual and mathematical models for P transport built by Sharpley and colleagues (Jones et al., 1984; Sharpley et al., 1984; Sharpley & Williams, 1990) and is still in use today. The fundamental relationship in these models, pioneered in Sharpley's early work (Sharpley et al., 1981; Sharpley & Ahuja, 1983), is that P in runoff is proportional to labile P. Our dataset of >600 soils and sediments across seven distinct watersheds followed this P lability framework to support model development and hypothesis testing.

Soils in this study stored enough labile P to sustain severe P loads for decades. Adding to this problem, legacy P also contributes to more stable P pools which, in turn, can slowly replenish the labile P pool—this dynamic, which several models already address, requires careful attention as we tackle legacy P stocks. Sediments were often more sorptive than nearby soils, leading to lower EPC_0_. At three sites, soils contained on average 32–90 mg kg^−1^ more labile P than sediments. Unifying soils and sediments in empirically based predictive relationships for either labile P or EPC_0_ will require predictors that characterize their biogeochemical differences; here, EPC_0_ was well‐predicted by labile P, BWI (a buffer capacity), DPS_ox_, and OC:(Al + Fe)_ox_. In soils under varying land uses and tillage regimes, labile P was clearly stratified by depth at all study locations, where topsoil (0–5 cm) had 1.3–3 times more labile P than soil beneath (5–15 cm), although total P was much less stratified (1–1.7). Overland flow P losses, particularly dissolved P, are at greater risk when labile P is concentrated in the surface layer.

Using the largest dataset—to our knowledge—of labile P (an extensive quantity), EPC_0_ (an intensive variable), and BWI (a buffer capacity), we established a strong nonlinear relationship, which generalizes well across all sample types, parent materials, and more factors studied here. Our results support the hypothesis that labile P drives the intensity of exchangeable P, whose concentration relative to ambient P concentrations can dictate the direction, removal versus release, of P sorption processes in the environment. However, this quantity–intensity relationship intensifies, or shifts toward greater solution P concentrations, when the buffer capacity is less and sorption sites are more occupied. This general relationship for P lability will improve our ability to predict P losses at the watershed and regional scale in landscapes with legacy P sources. Specifically, as several models predict dissolved P losses based on the connection between soil or sediment solution P and flow, it is critical to accurately translate the labile P quantity to P intensity. With better predictions, we may have greater insight into the efficacy of mitigation strategies. Empirical and modeling studies are needed to tailor conservation practices, old and new, to the legacy P challenge.

Sharpley's decades of research into the topics discussed here provided the framework for our understanding of legacy P. Generations now and future need to further his endeavor to repair the cycling of P in the environment and protect our waters.

## AUTHOR CONTRIBUTIONS

**Zachary P. Simpson**: Conceptualization; data curation; formal analysis; investigation; methodology; project administration; software; visualization; writing—original draft; writing—review and editing. **Joshua Mott**: Conceptualization; investigation; project administration; writing—original draft; writing—review and editing. **Kyle Elkin**: Data curation; investigation; methodology; resources; validation; writing—review and editing. **Anthony Buda**: Investigation; writing—review and editing. **Joshua Faulkner**: Investigation; writing—review and editing. **Cathleen Hapeman**: Investigation; writing—review and editing. **Greg McCarty**: Investigation; writing—review and editing. **Maryam Foroughi**: Investigation; writing—review and editing. **W. Dean Hively**: Investigation; writing—review and editing. **Kevin King**: Investigation; writing—review and editing. **William Osterholz**: Investigation; writing—review and editing. **Chad Penn**: Investigation; writing—review and editing. **Mark Williams**: Investigation; writing—review and editing. **Lindsey Witthaus**: Investigation; writing—review and editing. **Martin Locke**: Investigation; writing—review and editing. **Ethan Pawlowski**: Investigation; writing—review and editing. **Brent Dalzell**: Investigation; writing—review and editing. **Gary Feyereisen**: Investigation; writing—review and editing. **Christine Dolph**: Investigation; writing—review and editing. **David Bjorneberg**: Investigation; writing—review and editing. **Kossi Nouwakpo**: Investigation; writing—review and editing. **Christopher W. Rogers**: Investigation; methodology; writing—review and editing. **Isis Scott**: Investigation; writing—review and editing. **Carl H. Bolster**: Methodology; writing—review and editing. **Lisa Duriancik**: Conceptualization; funding acquisition; project administration; writing—review and editing. **Peter J. A. Kleinman**: Conceptualization; funding acquisition; methodology; project administration; supervision; writing—original draft; writing—review and editing.

## CONFLICT OF INTEREST STATEMENT

The authors declare no conflicts of interest.

## Supporting information



Supporting Material

Supporting Material

## Data Availability

All data described in this paper alongside R code to reproduce our analyses are available through Ag Data Commons: https://doi.org/10.15482/USDA.ADC/25892602.v1.
