# Supplementary material for: Phosphorus lability across diverse agricultural contexts with legacy sources
Source: J Environ Qual. 2024 Sep 29;54(4):851–69. doi: 10.1002/jeq2.20632 (PMC12265865; doi:10.1002/jeq2.20632)
Supplement: Supplementary file 1 — Supporting Material [file JEQ2-54-851-s002.pdf]

Extraction of Labile Phosphorus by Anion Exchange Membrane Incubation Method

Apparatus/Materials

- Type 1 anion exchange membrane
- Ultrapure water
- Ammonium Chloride
- Moisture balance
- 50 ml polypropylene tubes
- End over end shaker
- Balance or 2 g soil scoop
- Polypropylene analysis tubes

Principle

Ion exchange materials can be viewed as competitive exchangers with those soil solids[1] that are in dynamic equilibrium with the soil solution dissolved species. In the case of orthophosphate, at a relatively acid pH range (4.3-5.0), $H_2PO_4^-$ is transferred via the soil solution from the soil solid phase to the ion exchange material. The reaction is simple exchange of adsorbed Cl^- for other anions in solution. In contrast, the equilibrium reaction of $H_2PO_4^-$ with metal oxide coated materials (either Fe oxide coated filter papers or Al oxide coated resin) can be characterized as surface precipitation and adsorption via ligand exchange (Menon et al., 1990). This reaction is essentially irreversible, although anions like selenate, arsenate, and organic acids have been shown to compete with phosphate sorbed to Fe and Al oxyhydroxides (Traina et al., 1986). The resultant functional model for exchange resins relates to soil solution P dynamics. Since the mechanism for resin materials is ion exchange, there will be competition between $H_2PO_4^-$ and other anions at the resin sorption surface, particularly if other anion activities are high.

Over time, anion exchange materials will behave as either sinks or exchangers for P depending on: (i) the intrinsic anion exchange capacity of the resin material; (ii) the amount of time in contact with the soil; and (iii) the soil's P retention capacity. Throughout the literature, resin materials are described as infinite sinks, probably because their exchange capacities remain large across the study period or the soils' P retention capacities are low enough to minimize competition for P between the resin and soil solid phase.

In general, then, most anion exchange resins react rapidly with $H_2PO_4^-$, and the rate of sorption is limited by the rate of desorption or dissolution in the case of agitated systems, and by pore and film diffusion in the case of in situ resin placement.

Comments

Anion exchange membranes (AEMs) must be pre-saturated in warm solution (35-40 °C) solution for at least 24 hours and rinsed with water prior to use. AEMs should be stored under aqueous medium to keep them hydrated. Typical applications of AEMs use type 1 anion exchangers which are quaternary triethylamines. These resins, when in the chloride form, are relatively stable. Typical AEMs have an ion exchange capacity of 2-3 mol_c/kg dry, and have a high electrostatic resistance to cations.

Though there are numerous AEMs commercially available, one with high physical and chemical durability should be used.

[1] Soils are mentioned throughout this protocol but the same principles also apply to sediments.

Procedure

Anion exchange membrane preparation

1. Cut un-hydrated (dry) membrane strips into 1 inch squares (~6.4 cm^2).
2. Place squares in beaker of 0.5 M NaCl and lightly stir for 24 hours.
3. Rinse membranes with water and return to another beaker containing water.
4. Lightly stir membranes for an additional 24 hours, or until fully hydrated.

Analysis preparation

1. Weigh 2 g of <2 mm sieved soil in a 50 mL centrifuge tube and add 40 mL of water.
2. Place 1 anion exchange membrane square in the soil suspension and transfer to shaker. Agitate for 24 hours at 50 oscillations per minute.
3. Transfer membrane to a 50 mL tube containing 20 mL of 1M NH_4Cl and leave for 24 hours.
4. As necessary, dilute salt solution to appropriate concentration prior to P analysis.
5. Weigh 2 g of soil on moisture balance to get the correct moisture content for calculation below.

Calculation

$$\text{Labile P (mg kg}^{-1}\text{)} = \frac{\text{Sample conc. (mg L}^{-1}\text{)} * \text{Extract volume (L)} * \text{(dilution factor)} * 1000}{\text{Sample mass (g d.w.)}}$$

Sources

*Cooperband, L.R. and Logan, T.J. (1994). Measuring In Situ Changes in Labile Soil Phosphorus with Anion-Exchange Membranes. *Soil. Sci. Soc. Am. J.* 58: 105-114.

Menon, R.G., Chien, S.H., Hammond, L.L., Arora, B.R. (1990). Sorption of Phosphorus by the Iron Oxide Impregnated Filter Paper (P_i soil test) Embedded in Soils. *Plant Soil* 126:287-294.

Traina, S.J., Sposito, G., Hesterberg, D., Kafkafi, U. (1986). Effects of pH and Organic Acids on Orthophosphate Solubility in an Acidic, Montmorillonitic Soil. *Soil. Sci.Soc.Am. J.* 50:45-52.
