# Supplementary material for: Phosphorus lability across diverse agricultural contexts with legacy sources
Source: J Environ Qual. 2024 Sep 29;54(4):851–69. doi: 10.1002/jeq2.20632 (PMC12265865; doi:10.1002/jeq2.20632)
Supplement: Supplementary file 2 — Supporting Material [file JEQ2-54-851-s001.pdf]

Supplementary Information for '**Phosphorus lability across diverse agricultural contexts with legacy sources**'

Contents

Soil and sediment sampling protocols 2

Laboratory QA/QC 4

Estimating EPC_0 5

- Selecting the background matrix 5
- Selecting P concentrations 5
- Statistically pooling information to estimate EPC_0 6

Supplementary Results 9

- General soil and sediment chemistry 9
- Time since last P application and other land management effects 13
- Phosphorus stratification in soils and variation across land use 17
- Additional labile P stocks 18
- Generalized additive model for EPC_0 19

References 20

Soil and sediment sampling protocols

Table S1. Specifics of soil and sediment sampling at each of the seven study watersheds.

Watershed	Guiding hypotheses	Rationale for sampling	Other notes
**Lake Champlain**	(i) Sediment-bound P migrates from topographic high points to depressional areas within fields<br>(ii) Elevated soil P concentrations in depressions provide a surface runoff P source during 'fill and spill' occurrences<br>(iii) Elevated soil P concentrations in depressions become a subsurface runoff P source in tile drained fields	Closed depressions were selected within the study watersheds for sampling. An equal combination of tile drained and undrained fields were sampled. For each selected depression, two locations (transect endpoints) were sampled across an elevation gradient transect extending from the lowest point of the depression to the local high-point surrounding the depression. Control samples in adjacent forested areas not previously cultivated were also collected. Sediment samples were collected within stream channels at locations of monitoring stations in study watershed.	
**Le Sueur River (Upper Mississippi)**	(i) Subsurface drainage dissolved P losses are important in this region; (ii) P loss potential will be less for adjacent, unmanaged reference soils; (iii) Near channel environments (i.e., riparian areas, ditch and stream banks and bluffs, ravines, ditch and stream channels) are important potential areas for storage and transport of legacy P	Sampled along longitudinal gradients from farm fields to ravines, ditch and river banks; fields were subsurface (tile) drained with a history of hog manure application; nearby—P loss data were available for both subsurface and surface loss pathways; field site was within a watershed with substantial streambank sediment and P losses	Ditch samples had a high proportion of sand, and may not be representative of ditch banks across the watershed
**Beasley Lake (Lower Mississippi)**	Upslope soils tend to be coarser textured because, over time, finer material have eroded and been transported downslope along with nutrients and organic constituents. Therefore, soil test P and labile P tend to be higher downslope due to higher clay and organic C contents. For the same reason, sediments deposited in ditches and wetlands may be higher in clay and organic C and therefore higher in labile and soil test P.	Targeted surface flow paths across arable portion of watershed leading to the lake to quantify effects of soil type and flow path on legacy P sources; additional samples from upslope forested areas to downslope in riparian forested wetlands in a path leading to the lake, gives a contrast in land use; sediments were from drainage ditches, sediment retention pond, and lake to characterize eroded soil P dynamics	Some of the samples in wetland area on east side of lake are best characterized as hydric soils. Conventional tillage involved disking and bed formation in fall after harvest, and reformation of beds (sometimes preceded with further disking) in spring.
**Snake River Basin**	(i) Soil test P is higher and more stratified in sprinkler-irrigated fields compared to furrow irrigated fields.<br>(ii) Furrow-irrigated fields have higher labile P	Targeted top and bottom areas of irrigated commercial and research fields to quantify effects of type of irrigation (sprinkler versus furrow) and distance travelled by runoff on legacy P sources; sediment samples	Included fields with long-term manure applications.

	concentrations and are primary sources of P in irrigation return flow.<br>(iii) Soil test P concentrations in furrow-irrigated fields are higher in the bottom areas of the field, where deposition of furrow sediments occur.<br>(iv) Sediment P concentrations in return flow channels are lower compared to field soil P concentrations.	were collected from return flow channels to help characterize P dynamics in these often-overlooked streams.	
**Western Lake Erie Basin**	(i) P accumulation in closed depressions will be greater than hillslope contributing areas.<br>(ii) Soil test P and labile P will be greater in upland agricultural soils compared to upland forested areas and ditch sediment.	Targeted paired sampling of closed depression bottoms (i.e., lowest elevation) and hillslope contributing areas located in four fields and forested areas of a headwater watershed. Ditch bank and channel sediments collected along the length of the channel.	Site-specific results published in Mumbi et al. (2024)
**Mahantango Creek (Upper Chesapeake)**	(i) Soils in hydrologically active areas will have lower P concentrations vs. those in hydrologically inactive areas.<br>(ii) Sediment concentrations of P will be highest at the outlets of subcatchments with higher effective soil P status.	Soil sampling was designed to assess critical source areas of P loss across the watershed. Twenty paired transects were established that best captured the distribution of soils, management, and hydrological conditions in the watershed. For each transect pair, one followed a hydrologically active hillslope hollow, while the adjacent transect followed a hydrologically inactive ridgeline. Sediments were sampled at the outlets of major subbasins representing a range of catchment effective soil P statuses.	
**Chesapeake Bay (Lower Chesapeake)**	Soils receiving frequent manure application from animal operations have higher legacy P. Individual field management has major influence. Soils in more poorly drained landscape positions (depressional areas) are expected to have higher clay and P content. Farmers will preferentially enhance overland flow processes on poorly drained soils which will also be likely critical source areas for P delivery to ditch and stream networks.	Used LiDAR-derived topographic metrics to characterize poorly drained landscape positions, topographic controls on soil test P, and farmer management practices that enhance overland flow. Distributed intensive soil sampling within balanced categories of topographic openness.	Fields often contain prior converted croplands that were depressional wetlands before drainage. The areas are poorly drained, accumulate P, and are often enhanced by farmers to promote overland flow for water removal.

Laboratory QA/QC

Sample analyses were conducted using standard methods as described in soil and water analysis methods manuals (APHA, 2017; Soil Survey Staff, 2022) unless noted otherwise. All analytical procedures followed calibration in matrix-matched solutions and unknowns were bracketed by a method test blank, calibration blank check, and quality control standards (two in-house and one 3rd party). Within each set of unknown samples, at least two samples were duplicated in the analysis to determine within-sample variability (within 1%); for extraction methods, two other samples were analyzed twice (i.e., for the same extract, but analyzed twice on the instrument) to determine instrument precision (within 0.25%). Instrument detection limits were determined by the mean of 10 consecutive blanks plus the standard deviation times the critical *t*-value for the 99th percentile (3.25). Method detection limits were determined by the lowest reproducible value that could be repeatedly measured from a matrix matched, laboratory fortified blank (see dataset for more information).

With each batch of analyses, up to three reference soils from the North American Proficiency Testing program (NAPT) were included. For the analyses available in NAPT (e.g., pH, total carbon, Mehlich-3), results for the reference soils were within the suggested range of NAPT's interlaboratory median value ± 2.5 times the median absolute deviation.

Estimating EPC_0

We sought to determine EPC_0 for the nearly 600 soil and sediment samples collected in the project. Due to the large number of samples, and the fact that EPC_0 determination is relatively intensive, we faced the challenge of limited analytical capacity in estimating these EPC_0 values. Protocols were developed to overcome this challenge, among others, which are described here.

Selecting the background matrix

Selection of an appropriate solution background matrix for the equilibrations is imperative, as ionic strength, specific cation concentrations (e.g., Ca^{2+}), and more can influence the EPC_0 (Barrow and Shaw, 1979; Lucci et al., 2010; Simpson et al., 2021). Simpson et al. (2021) synthesized the literature around this point, suggesting that, for most samples collected in the current study, a solution with 0.5 mM $CaCl_2$ and 0.5 mM NaCl (ionic strength of 2 mM) would be optimal. However, the Snake River samples originate from a carbonate-rich lithology (e.g., see total Ca in Table 3 in main text), meaning solution concentrations of cations, particularly Ca^{2+} are significantly greater than for the other six sites. For these samples, we used a solution with 1.5 mM $CaCl_2$ and 0.5 mM NaCl. This solution closely mirrors the ionic strength and Ca^{2+} activity of environmental waters in the area and furthermore will suppress excess surface dissolution reactions (e.g., of various phosphate minerals such as hydroxyapatite or phosphate co-precipitated with carbonates) during equilibrations (Lindsay and Moreno, 1960).

To this point, we applied the 0.5 mM $CaCl_2$ solution to the Snake River samples in a side experiment. The results in Figure S1 illustrate that the weaker background shifted the P sorption curve to the right for most samples at Snake River, sometimes by a large margin. As a consequence, EPC_0 for these data would be greater than for the stronger background. Interestingly, the 'QUIN' reference soil was highly sensitive to the choice of background while the other two reference soils were not (although the stronger matrix did seem to shift sorption up, in line with theory that the electric double layer for these soils is suppressed, thus favoring more sorption). Although uncertain, this suggests that exchangeable P in the 'QUIN' soil may be driven more by precipitation/dissolution reactions rather than ligand exchange (more likely the case in the 'HAG' and 'HUB' reference soils), despite lacking a high pH (6.35 in H_2O).

Selecting P concentrations

While it is preferable to use several initial P (as PO_4) concentrations and multiple replicates when estimating EPC_0, we were constrained due to the large number of samples involved. As a compromise, we restricted the equilibrations to three or four per sample to allow EPC_0 estimates across all soils and sediments in the study. For each batch of EPC_0 (typically all the soils and sediments from a given site), we initially applied 0 and 0.25 mg P L^{-1} as the initial concentration. Judging by the final solution concentrations, we then either ran a 2.5 mg P L^{-1} concentration (if no adsorption had occurred by 0.25 mg P L^{-1}) or a lower concentration (usually 0.025 mg P L^{-1}). In some extreme cases, we had to include even higher concentrations to ensure the sample exhibited adsorption.

For the equilibrations, we only used one replicate at each concentration. While less than ideal given the typical variation in these measurements, we were able to leverage the large number of samples involved at each site to improve EPC_0 estimates (see section 'Statistically pooling information to estimate EPC_0' below). After inspecting the initial results, we opted to add additional replicate equilibrations to roughly 150 samples (mostly just one replicate per sample) to improve EPC_0 estimates. These samples involved either experimental error or initial concentrations far from the likely EPC_0, both of which preclude good estimation. These re-runs were selected via visual inspection; as before, frozen samples were thawed to provide a reasonably fresh sample for equilibration.

Additionally, to improve the statistical model and to make the most of the limited number of replicates, we applied a weighting function to the data so that extreme adsorption and desorption points have less leverage on the EPC_0 estimate. For each sorption point, q (mg P kg^{-1}), the weight was $\exp(-0.1\,|q|)$. This weighting function emphasizes (weights >0.5) sorption values between ±7 mg P kg^{-1}.

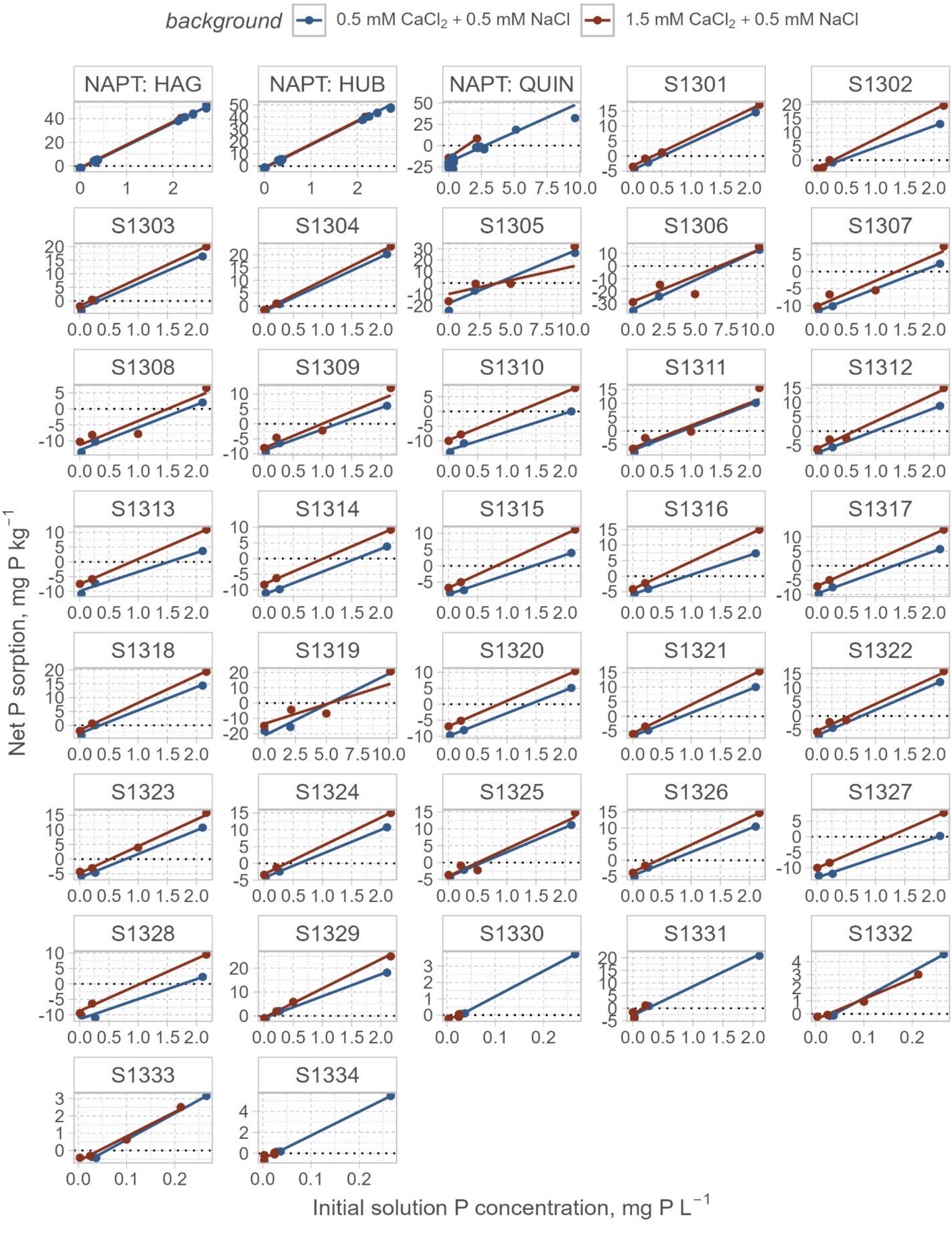

Figure S1. Effect of solution background on sorption measurements for EPC_0 in the Snake River samples (ID's beginning with "S") and three NAPT reference soils (top-left three plots). Regression lines are merely illustrative and do not reflect the final EPC_0 estimate (e.g., no statistical pooling is employed here).

Statistically pooling information to estimate EPC_0

Traditionally, EPC_0 is estimated on a per-sample basis using replicate equilibrations all from the same soil or sediment via a regression of some sort (Simpson et al., 2021). Given sufficient replicates and good targeting of initial P concentrations, this yields an accurate estimate. Here however, as hinted above, we applied statistical pooling to leverage the information

across all the equilibrations and thus improve each sample's EPC_0 estimate. We applied a multilevel model via the 'brms' package (Bürkner, 2017) of the form:

$$q_{i,j} \sim N(\mu_i, \sigma)$$

$$\mu_i = \beta_{1,j}(c_i - 10^{\beta_{0,j}})$$

$$[\beta_{0,j}, \beta_{1,j}] \sim MVN([\overline{\beta_0}, \overline{\beta_1}], R, [\sigma_0, \sigma_1])$$

where $q_{i,j}$ is the sorption value (mg P kg^{-1}) for replicate i of sample j, which is weighted appropriately (see above); c_i is the initial P concentration (mg P L^{-1}) for replicate i; μ_i is the conditional mean response, which is effectively a linear function of c_i but is rewritten as nonlinear to allow EPC_0 itself (represented here as $10^{\beta_{0,j}}$; mg P L^{-1}) to be estimated as a parameter while β_1 is the sorption slope (mg P kg^{-1} per mg P L^{-1}, or L kg^{-1}); the parameters $\beta_{0,j}$ and $\beta_{1,j}$ are assumed to come from a common multivariate normal distribution with means at the site level of $\overline{\beta_0}$ and $\overline{\beta_1}$, standard deviations σ_0 and σ_1, and a correlation structure between the varying effects R. Note that the function for μ_i is written with a back-transformation of the $\log_{10}$ of the EPC_0 ($\beta_{0,j}$); this not only improved posterior sampling but naturally avoided sampling in the unrealistic space of negative EPC_0.

This model allows data from related samples to inform each other regarding sorption characteristics through partial pooling of information (McElreath, 2020). Since the model assumes that the samples j are statistically exchangeable, we fitted the model on a site by site basis: the rationale is that soils and sediments from the same general region share similar lithologies, clay mineralogy, and other characteristics related to P sorption. Further differences in samples at the same site are handled with basic covariates, discussed below.

We modified this base model by including other sample characteristics as linear predictors for the parameters $\beta_{0,j}$ and $\beta_{1,j}$. We included here Mehlich-3 P (P_{M3}), degree of P saturation (via oxalate extraction, DPS_{ox}), Olsen P (P_{Ols}; only for Snake River for concern of alkaline soil chemistry; ultimately, we found little difference between using either P_{M3} or P_{Ols}), and sample type (sediment vs. soil) as predictors. Our rationale was that samples with greater P exposure (and hence greater soil test P and DPS_{ox}) would have greater EPC_0; further, all else equal, more P exposure would lower the sorption slope (i.e., we expect $\beta_{0,j}$ and $\beta_{1,j}$ to be correlated); lastly, differences between soils and sediments at each site could manifest through, e.g., particle size sorting and different redox regimes. For the continuous predictors, these were centered to have a mean of zero on a site basis (but not scaled) in order to ease prior specification.

We discuss here only four of the primary model formulas tested (labeled with the ID's of 0, 5, 6, and 7). A model with zero pooling (akin to a regression per each sample) performed poorly in tests and was excluded. Models with key predictor effects (soil test P or DPS_{ox}) on either β_0 or β_1 alone (not simultaneous) performed poorly, likely due to not recognizing the negative correlation between these two parameters (samples with greater EPC_0 also tend to have weaker sorption slopes). Priors were specified to be weakly informative to improve sampling (Table S2). For example, priors on the EPC_0 parameter emphasized values from the part-per-billion to the part-per-million range, reflecting the breadth of values in reviews such as Simpson et al. (2021). For the effect of sample type (soil vs. sediment), we made the prior unbiased (mean of zero); however, for predictors such as soil test P and DPS_{ox}, we suggested in our priors to expect a positive effect on β_0 but a negative effect on β_1 – specifying an expected zero effect did not alter posterior estimates in tests but did slow down sampling efficiency.

For each site and model, we employed 6 chains with 2500-5000 iterations each ($\geq$7500 post-warmup draws), which generally yielded bulk and tail effective sample sizes >1000 and R-hat $\leq$1.01.

Table S2. Description of priors used in multilevel models for EPC_0. The standard deviation parameters (σ) have a lower bound of 0. For prior distributions, N *is the normal (mean, standard deviation),* Exp *the exponential (rate),* Student *the Student-t (location, degrees of freedom, scale), and* LKJ *the Lewandowski-Kurowicka-Joe distribution. In `brms`, the default Student-t priors for σ use 3 degrees of freedom and a scale parameter dependent on the dataset. For priors where two distributions are mentioned, the first is the default and the second is a somewhat more informative prior used in cases to improve sampling efficiency. Small tweaks in these priors were used for different sites as needed.*

Model parameter	Predictor variable	Model number applicable	Prior distribution
β_0	Intercept	0, 5, 6, 7	*N*(-0.5, 0.5)
	Centered P_{M3}	5	*N*(0.004, 0.01)
	Sample type	5, 6, 7	*N*(0, 0.25)
	Centered P_{M3} × Sample type	5	*N*(0, 0.01)
	Centered DPS_{ox}	6	*N*(0.05, 0.1)
	Centered DPS_{ox} × Sample type	6	*N*(0, 0.01)
	Centered P_{Ols}	7	*N*(0.02, 0.1)
	Centered P_{Ols} × Sample type	7	*N*(0, 0.01)
β_1	Intercept	0, 5, 6, 7	*N*(10, 5)
	Centered P_{M3}	5	*N*(-0.02, 0.3)
	Centered DPS_{ox}	6	*N*(-0.25, 0.5)
	Centered P_{Ols}	7	*N*(-0.1, 0.5)
σ		0, 5, 6, 7	*Student*(0, 3, scale)
σ_0	Intercept	0, 5, 6, 7	*Exp*(5)
σ_1	Intercept	0, 5, 6, 7	*Student*(0, 3, scale) or *Exp*(1)
R		0, 5, 6, 7	*LKJ*(1) or *LKJ*(0.75)

When comparing the competing models for each site, we primarily used the leave-one-out cross-validation information criterion (LOO-CV) (Vehtari et al., 2017) via the `loo` package (Vehtari et al., 2023), posterior predictive checks, and visual checks of the resulting EPC_0 estimates against the raw data. Generally, we opted for the model with the lowest LOO-CV information criterion (LOO-IC) but in one case (Le Sueur, due to likely measurement error issues) used the 2nd best model (in terms of LOO-IC) based on visual checks.

Table S3. Chosen model for EPC_0 for each site. As a comparison to the simpler reference model 0, the difference in expected log pointwise predictive density for a new dataset (ELPD) is given; we follow the heuristic where differences of -4 or lower indicate substantial improvement in model performance (at least for the raw sorption data).

Site	Chosen model	Difference in ELPD to model 0
**Lake Champlain**	5	-15.3 (7.6)
**Le Sueur**	6	-1.4 (3.1)
**Beasley Lake**	6	-29.5 (3.8)
**Snake River**	5	-13.0 (4.3)
**W. Lake Erie**	5	-11.3 (3.2)
**Upper Chesapeake**	5	-24.9 (4.9)
**Lower Chesapeake**	6	-9.9 (5.7)

Supplementary Results

General soil and sediment chemistry

Extractable Fe by either oxalate or bicarbonate-dithionite (both 2 h extractions) were highly variable for each site (Figure S2) for most sites, especially for sediments, $Fe_{ox} > Fe_{BD}$ while U. Chesapeake favored $Fe_{ox} < Fe_{BD}$. This variation is likely linked to the different Fe mobilization processes for the BD (reductive dissolution) and oxalate (chelation and protonation) extractions (McKeague and Day, 1966; Jan et al., 2015) as well as the different mineralogies and reductive environments of each site. To this point, Figure S3 illustrates that sites generalized as having more illite/mica clays (U. Chesapeake, Snake River) favored more Al_{ox} and greater DPS_{ox} than other sites characterized as having more weathered clays (e.g. kaolinite). Of the P-reactive metal oxides studied here, Al_{ox} was the most strongly associated with clay concentration (ρ=0.71). Further, DPS_{ox} was strongly related to the Fe_{ox} (ρ=-0.67) and Fe_{BD} (ρ=-0.57) concentrations in general (for Al_{ox}, ρ=-0.23), suggesting that P saturation as measured with DPS_{ox} appears more sensitive to Fe than Al oxides. The Fe_{BD}:P_{BD} ratio and Fe_{BD} was more homogeneous across sites and clay concentrations, arguably due to a greater sensitivity to the redox regime of the soil or sediment (Peiffer et al., 2021; Smith et al., 2023).

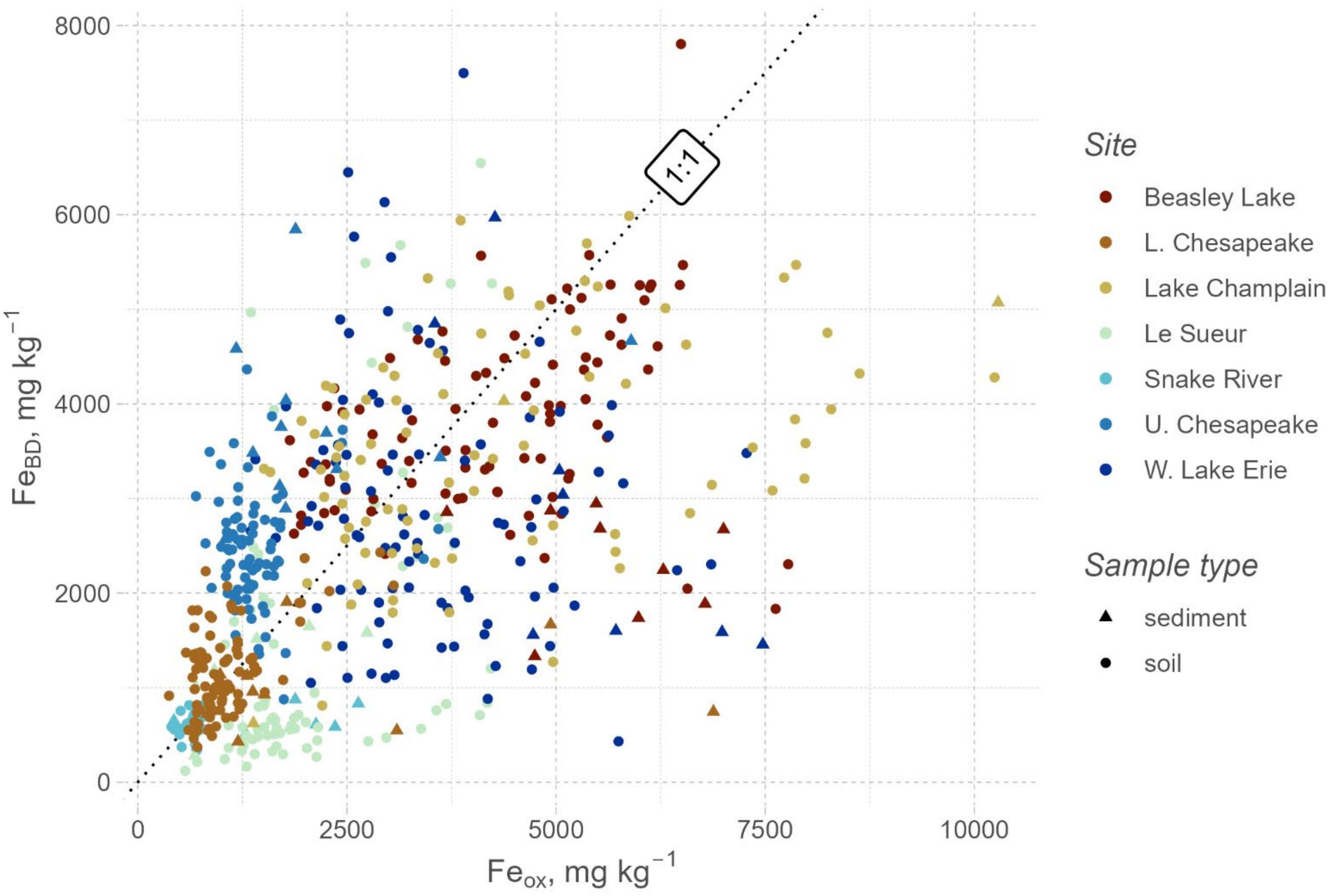

Figure S2.Comparison of soil and sediment iron (Fe) extractable by acid ammonium oxalate (Fe_{ox}) and by bicarbonate-dithionite (Fe_{BD}). Both extractions were for 2 h but field-moist samples (frozen) were used for bicarbonate-dithionite. While correlated, there is considerable variation between the extractions, which are nearly evenly split with 53% of the data having $Fe_{ox} > Fe_{BD}$.

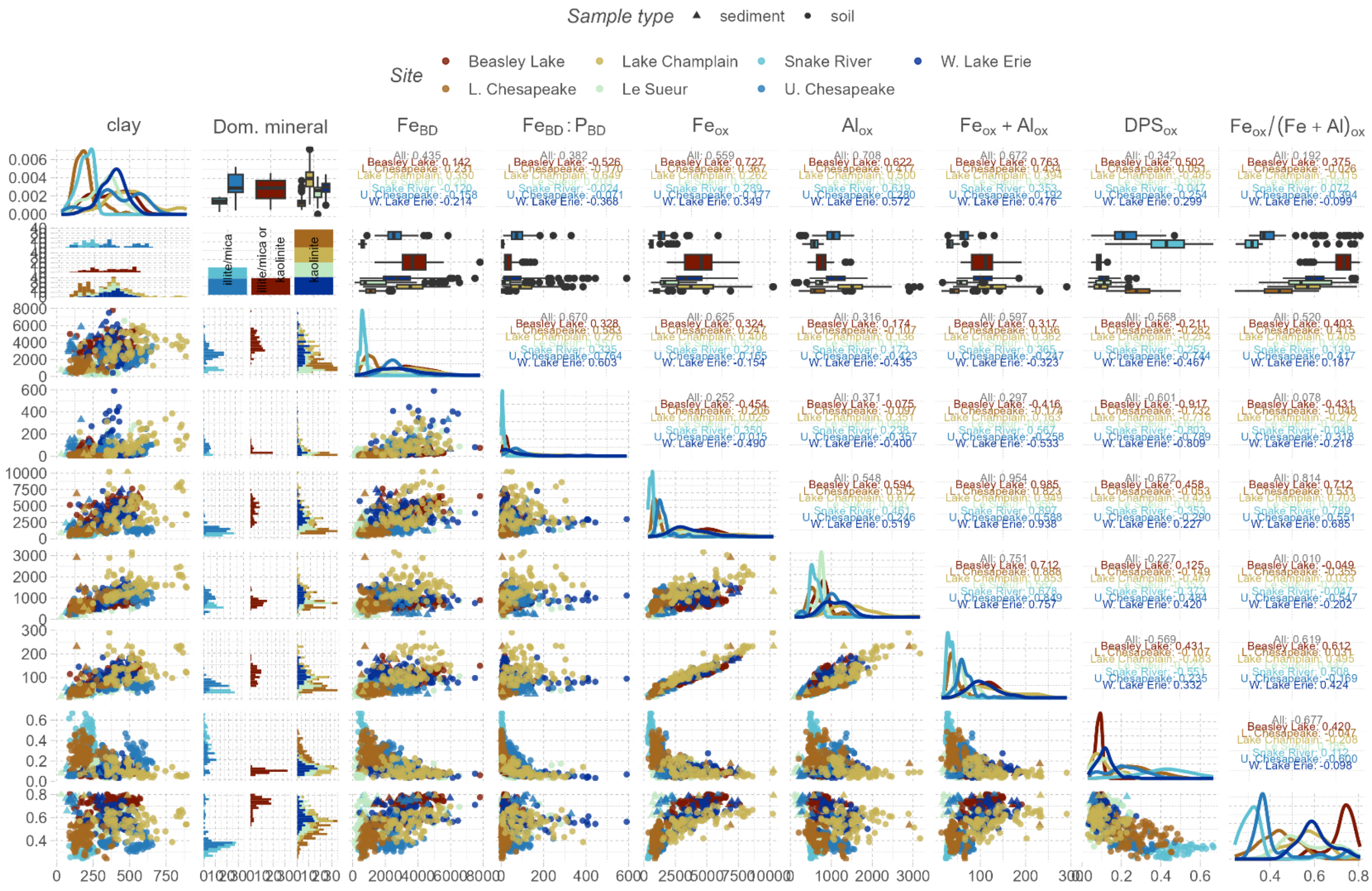

Figure S3.Correlation matrix for Fe, Al, and clay related variables across soils and sediments from the seven study sites. Values in upper triangle are Spearman's rho (ρ) correlations (across soils and sediments). See Table 2 (main text) for further details and units. Dominant clay mineralogy is sourced from (Ito and Wagai, 2017); the dominant clay minerals here were illite/mica (Snake River and U. Chesapeake), either illite/mica or kaolinite (Beasley Lake), or kaolinite (remaining sites). Note that elemental ratios or sums are calculated with concentrations in millimolar units. Indices for statistical significance are omitted here for clarity, but as a reference: $|\rho| \geq 0.197$ and $|\rho| \geq 0.338$ are the critical values at a null probability of 0.05 for sample sizes of 100 (max per site) and 34 (minimum sample size here, corresponding to frozen analyses at Snake River); most tests here are for sample sizes of 64 - 100.

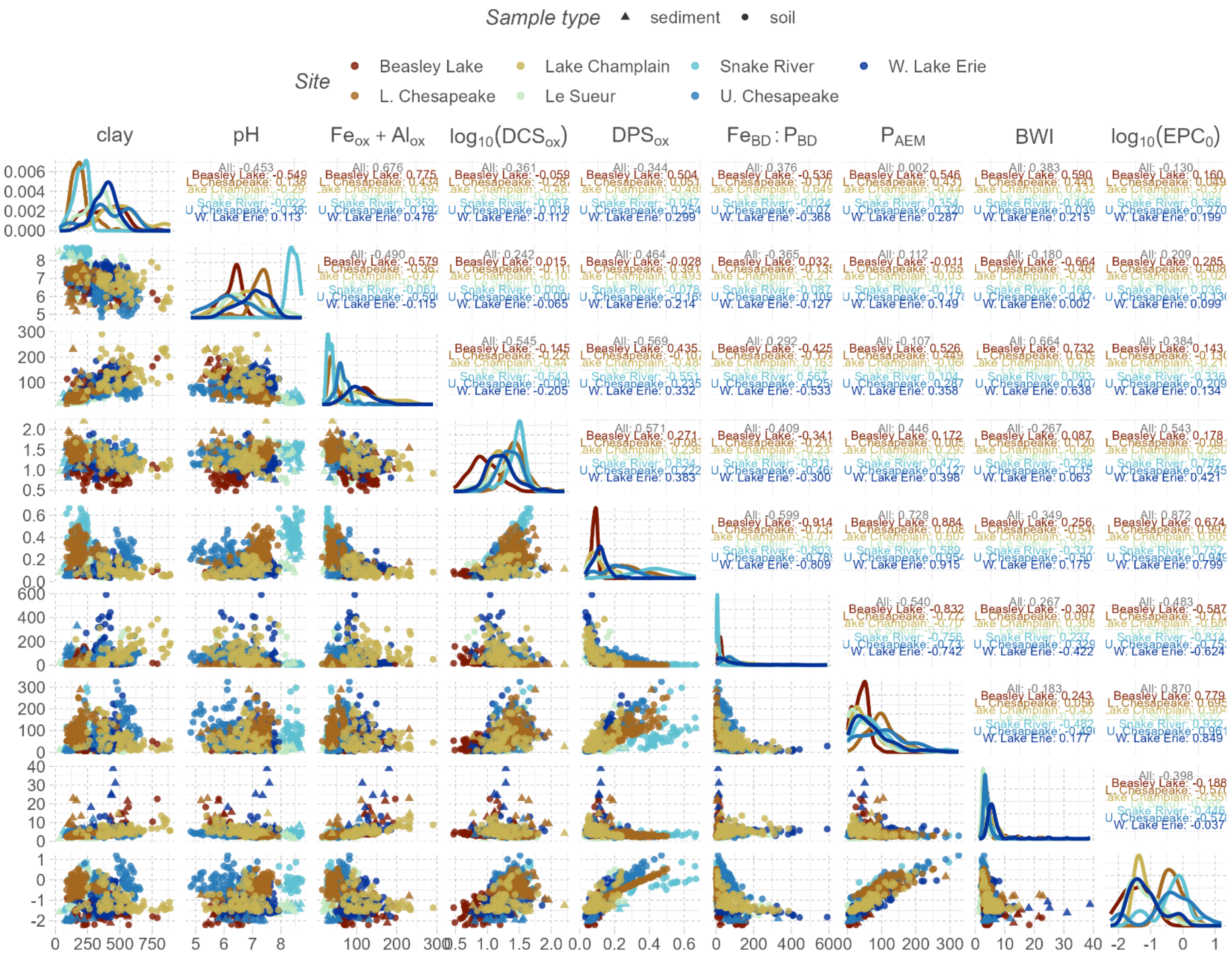

Figure S4.Correlation matrix of select physicochemical properties and the core P-specific variables of the study (labile P, EPC_0, and Bache-Williams index [BWI]) across all samples and seven study sites. Values in upper triangle are Spearman correlations (across soils and sediments). See Table 2 (main text) for further details and units. Indices for statistical significance are omitted here for clarity, but as a reference: $|\rho| \geq 0.197$ and $|\rho| \geq 0.338$ are the critical values at a null probability of 0.05 for sample sizes of 100 (max per site) and 34 (minimum sample size here, corresponding to EPC_0 at Snake River); most tests here are for sample sizes of 64 - 100.

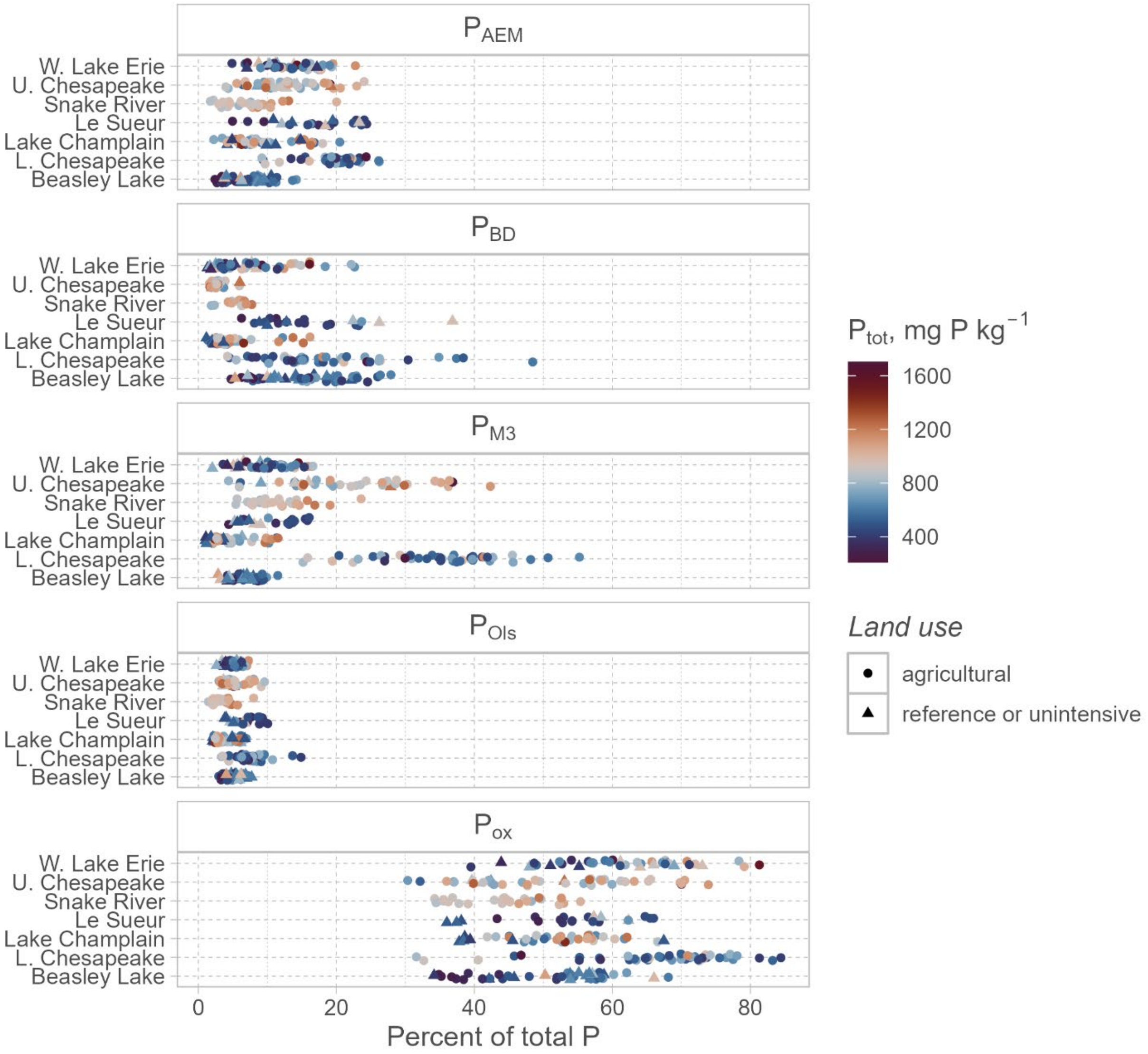

Figure S5. The percentage of total P (P_{tot}) present in various soil P extractions for topsoils (0 – 5 cm) under both agricultural and unintensive/reference land uses. Note that Snake River and L. Chesapeake only have samples from agricultural fields. P extractions (top to bottom) are anion-exchange membrane for 24 h (labile P), bicarbonate-dithionite (reductively soluble P), Mehlich-3 and Olsen P (soil test P), and acid ammonium oxalate (P complexed with Al, Fe, and Mn).

Time since last P application and other land management effects

Though we targeted sites characteristic of legacy P dynamics, some of the fields had recent P applications; the most recent being one field at the Snake River site (~6 months). However, across the project, we note no general trends (i.e., consistent across sites) with time since last application for the core P variables (Figure S6). While our study is not designed to estimate it, we did test for whether time since P application had any apparent effect via a model. The model included a monotonic trend (as time of last P application was uncertain in some cases, e.g., classed as >5 yr) as a varying effect over site. For all variables tested – P_{AEM}, EPC_0, BWI, DPS_{ox}, and soil test P – none exhibited a consistent trend (the 95% CI straddled 0 in all cases).

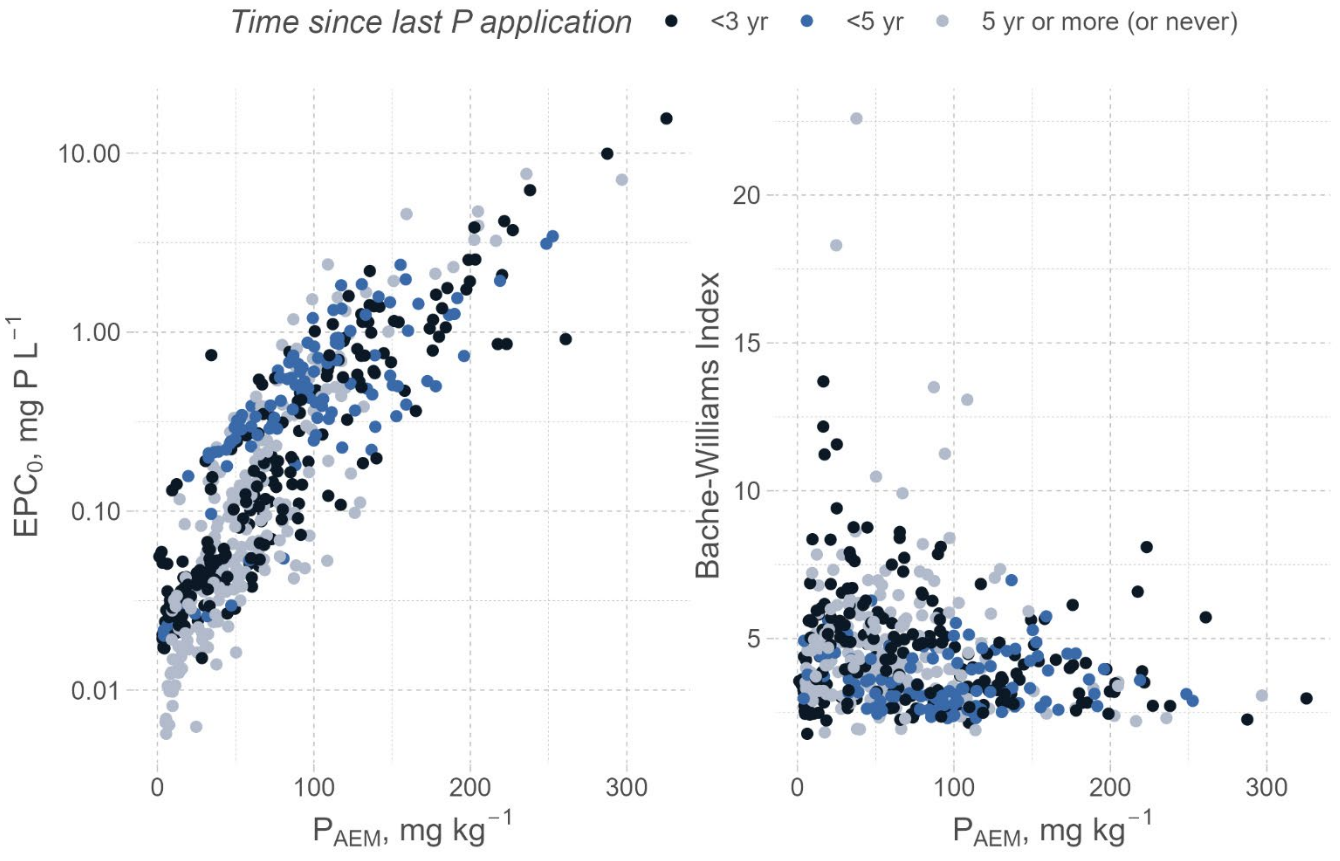

Figure S6. For soils in the project, time since last P application (all sources) had no apparent effect on core P relationships, including between (left) labile P (P_{AEM}) and EPC_0 or between (right) labile P and the Bache-Williams index (measure of P buffer capacity).Time since application was discretized to account for the uncertainty for some fields (e.g., records indicate last application was 6-8 yr ago).

We illustrate below in strictly broad terms the effect of land use for the five sites that sampled across diverse land uses, but note this is a coarse view of the sites' complexities. In Figure S7 and Figure S8, soil data for physicochemical and P-specific variables, respectively, are plotted for two simplified land use classes (reference or unintensive vs. agricultural). Generally, physicochemical properties are broadly comparable (notwithstanding varying sample sizes and other factors). P-specific variables were generally more intense under agricultural land uses compared to their nearby reference sites (e.g. medians were greater), but more site-specific information is required to probe this relationship. For example, model-based contrasts of P_{AEM} between the simplified land uses show highly uncertain effects (Figure S9). Further investigation at the site level is required to understand what impacts these various land uses may have on P properties.

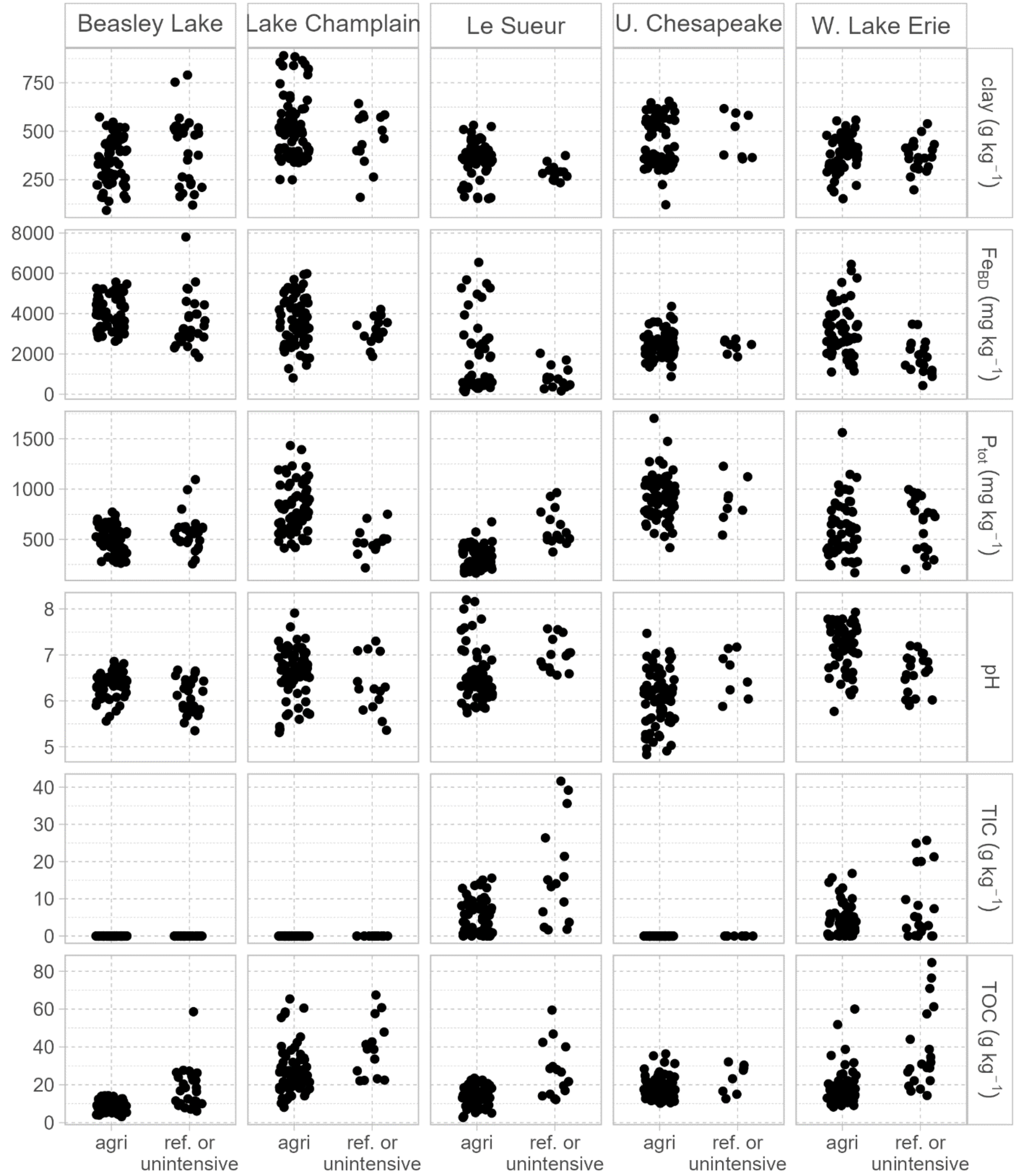

Figure S7. Comparison of select general properties in soils at the five sites with samples from both agricultural ('agri') and reference (ref.) or unintensive land uses. Note that the L. Chesapeake and Snake River sites only sampled from agricultural land use.

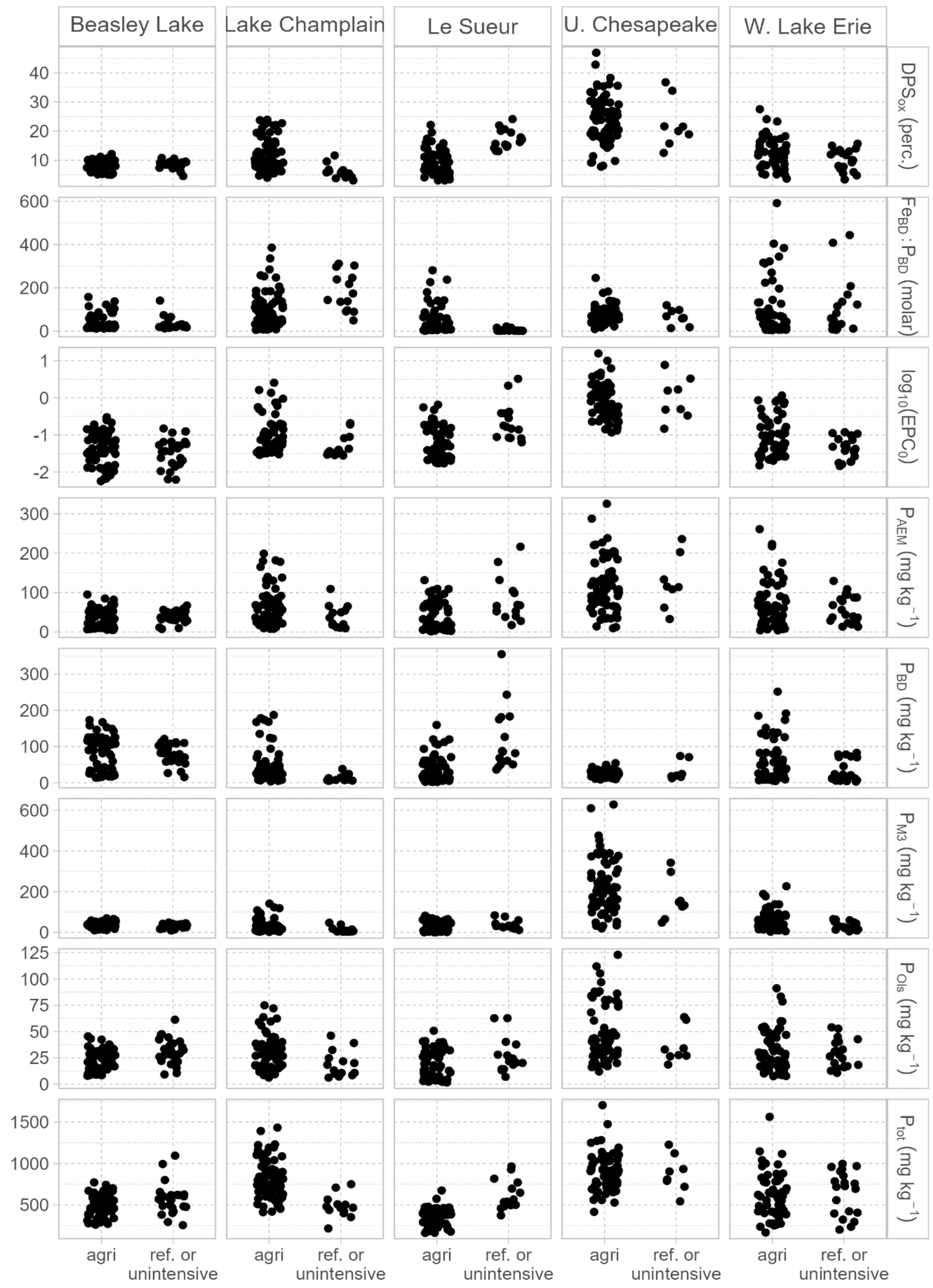

Figure S8. Comparison of select P-related properties in soils at the five sites with samples from both agricultural ('agri') and reference (ref.) or unintensive land-use. Note that the L. Chesapeake and Snake River sites only sampled from agricultural land use.

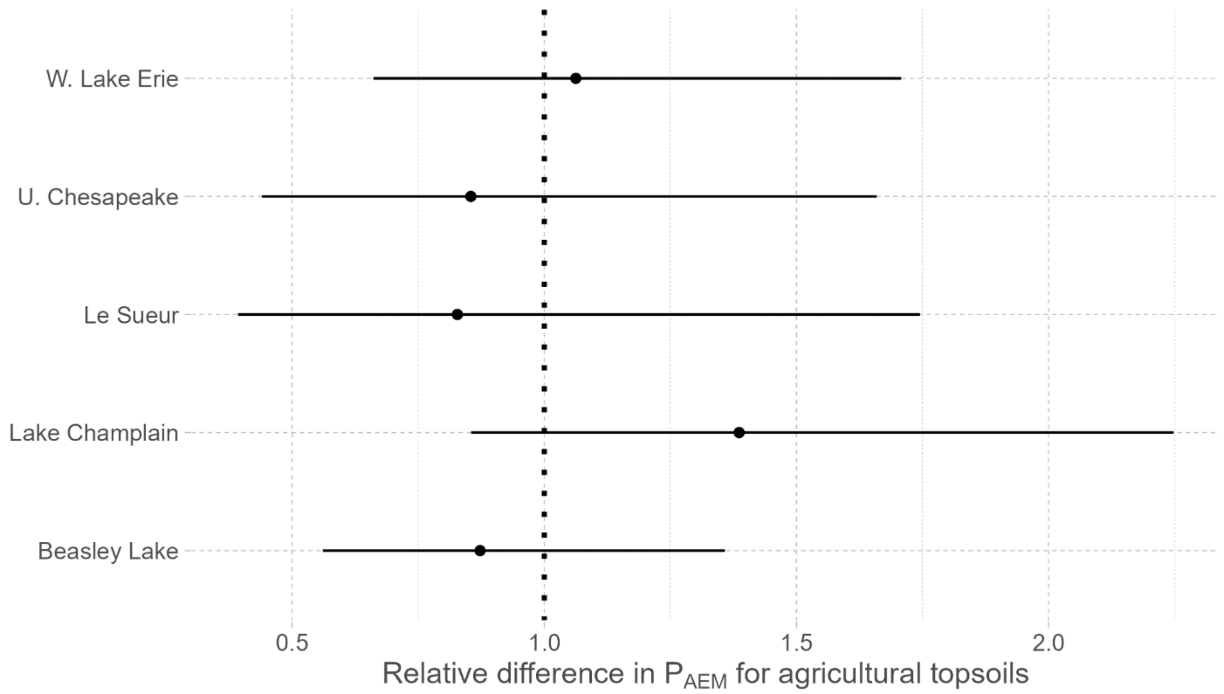

Figure S9. For topsoils (0 – 5 cm), the relative difference in labile P (P_{AEM}) concentrations between agricultural and reference/unintensive land uses; a value of 1 (dashed line) indicates no difference. Points are the mean and error bars are 95% confidence intervals. A model was fit to each site's data to account for site-specific relationships, and pH, clay, and ratio of total organic C to oxalate-extractable Al + Fe were covariates.

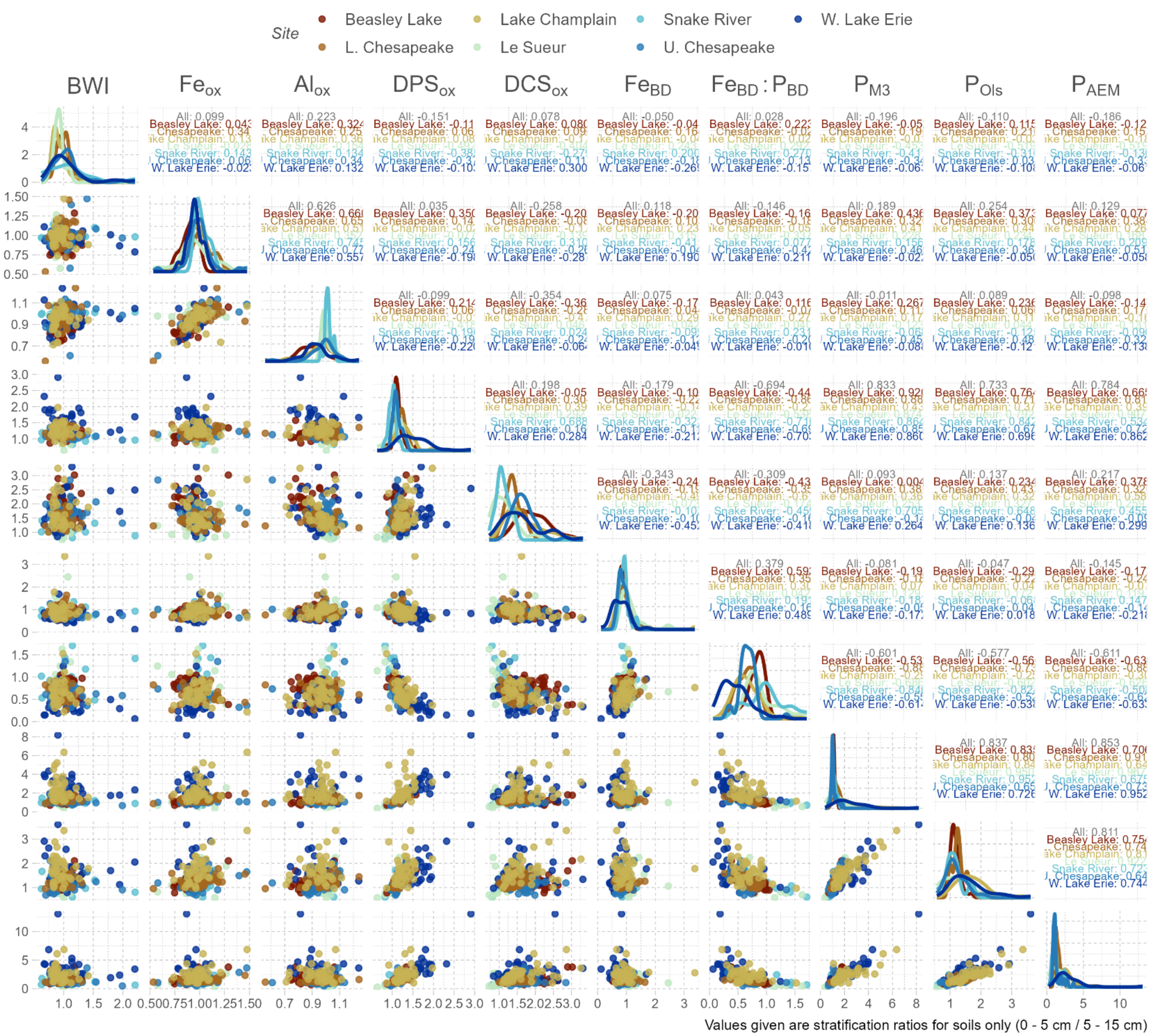

Figure S10. Correlation matrix of stratification ratios *for select soil P quantities and sorption-related characteristics. All values shown are stratification ratios of the corresponding variable for soils only (0 -5 cm divided by 5 – 15 cm). Values of 1 indicate no stratification; >1 indicates that the upper soil layer is greater. Values in upper triangle are Spearman correlations. See Table 2 (main text) for further details and units as well as Figure 2 (main text). Indices for statistical significance are omitted here for clarity, but as a reference: $|\rho| \geq 0.208$ and $|\rho| \geq 0.373$ are the critical values at a null probability of 0.05 for sample sizes of 89 (max per site) and 28 (minimum sample size here, corresponding to frozen samples at Snake River); most tests here are for sample sizes of 39 - 89.*

Additional labile P stocks

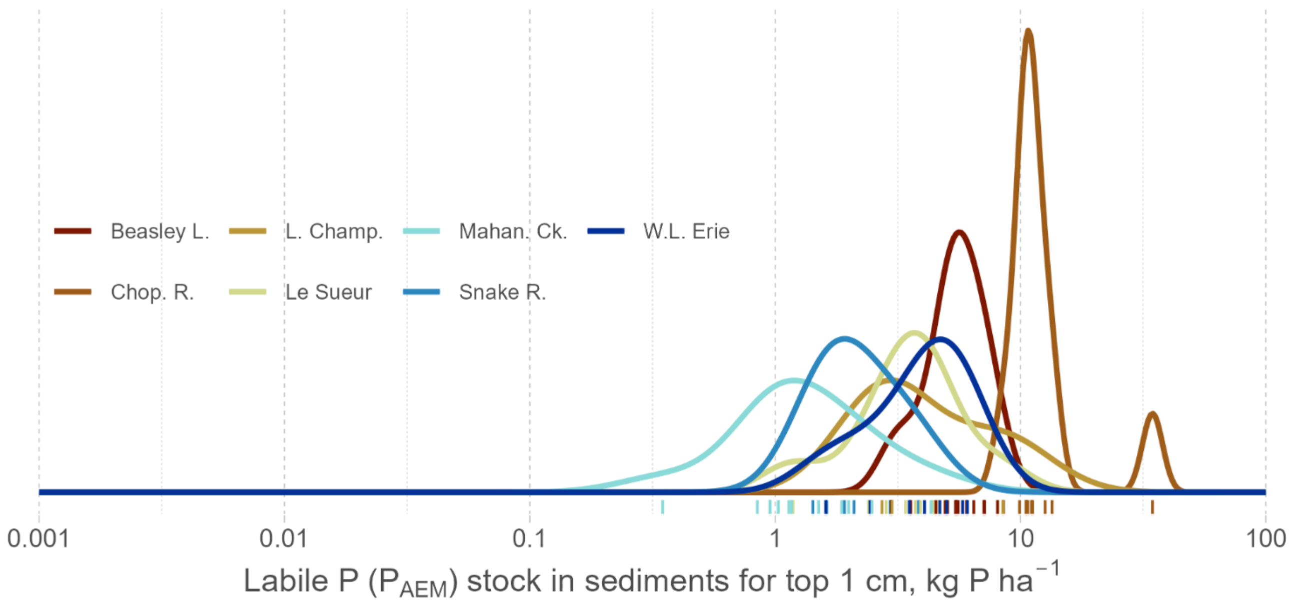

Figure S11. Probability densities for sediment stocks of labile P (P_{AEM}) across sites. As for Figure 3, the stocks of P_{AEM} (bottom) assume the top 1 cm of sediment and a bulk density of 1.2 g cm^{-3}.

Generalized additive model for EPC_0

Table S4. Model summary statistics for the generalized additive model (GAM) of EPC_0 as a function of labile P and related P buffer variables. The intercept is the only parametric term in the model and its estimates are given on the log (link) scale. The s() and te() terms denote smoothing spline and tensor product smooths, respectively. Their effective degrees of freedom describe the complexity of the smooth. The null hypothesis tested for either parametric coefficients (t statistic) or semiparametric smoothing spline functions (F statistic) is that they are zero; for splines, these are approximations; details are in Wood (2013). Note that smoothing spline effective degrees of freedom approach one as the smooth is penalized toward a straight line.

**Model term**	**Estimate (standard error)**	**Effective degrees of freedom**	***F* or *t* statistic**	**Probability(>|*F* or *t*|)**
**Intercept**	-1.91 (0.094)	1	-20.4	<2e-16
**te(log (P_{AEM}), BWI)**		19.2	32.4	<2e-16
**s(log(DPS_{ox}))**		1.63	43.7	<2e-16
**s(log(OC:$(Al+Fe)_{ox}$))**		2.02	11.1	<2e-16
**s(site)**		5.51	12.0	<2e-16

References

APHA. 2017. Standard Methods for the Examination of Water and Wastewater. 23rd Edition. American Public Health Association, Washington, D.C.

Barrow, N.J., and T.C. Shaw. 1979. Effects of Ionic Strength and Nature of the Cation on Desorption of Phosphate from Soil. J. Soil Sci. 30(1): 53–65. doi: 10.1111/j.1365-2389.1979.tb00964.x.

Bürkner, P.-C. 2017. brms: An R Package for Bayesian Multilevel Models Using Stan. J. Stat. Softw. 80(1): 1–28. doi: 10.18637/jss.v080.i01.

Ito, A., and R. Wagai. 2017. Global distribution of clay-size minerals on land surface for biogeochemical and climatological studies. Sci. Data 4(1): 170103. doi: 10.1038/sdata.2017.103.

Jan, J., J. Borovec, J. Kopáček, and J. Hejzlar. 2015. Assessment of phosphorus associated with Fe and Al (hydr)oxides in sediments and soils. J. Soils Sediments 15(7): 1620–1629. doi: 10.1007/s11368-015-1119-1.

Lindsay, W.L., and E.C. Moreno. 1960. Phosphate Phase Equilibria in Soils. Soil Sci. Soc. Am. J. 24(3): 177–182. doi: 10.2136/sssaj1960.03615995002400030016x.

Lucci, G.M., R.W. McDowell, and L.M. Condron. 2010. Evaluation of base solutions to determine equilibrium phosphorus concentrations (EPC<inf>0</inf>) in stream sediments. Int. Agrophysics 24(2): 157–163.

McElreath, R. 2020. Statistical Rethinking: A Bayesian Course with Examples in R and Stan. 2nd ed. Chapman and Hall/CRC, New York.

McKeague, J.A., and J.H. Day. 1966. Dithionite- and oxalate-extractable Fe and Al as aids in differentiating various classes of soils. Can. J. Soil Sci. 46(1): 13–22. doi: 10.4141/cjss66-003.

Mumbi, R.C.K., M.R. Williams, C.J. Penn, and J.J. Camberato. 2024. Accumulation of soil phosphorus within closed depressions of a drained agricultural watershed. Soil Sci. Soc. Am. J. n/a(n/a). doi: 10.1002/saj2.20671.

Peiffer, S., A. Kappler, S.B. Haderlein, C. Schmidt, J.M. Byrne, et al. 2021. A biogeochemical–hydrological framework for the role of redox-active compounds in aquatic systems. Nat. Geosci. 14(5): 264–272. doi: 10.1038/s41561-021-00742-z.

Simpson, Z.P., R.W. McDowell, L.M. Condron, M.D. McDaniel, H.P. Jarvie, et al. 2021. Sediment phosphorus buffering in streams at baseflow: A meta-analysis. J. Environ. Qual. 50(2): 287–311. doi: 10.1002/jeq2.20202.

Smith, G.J., R.W. McDowell, K. Daly, D. Ó hUallacháin, L.M. Condron, et al. 2023. Factors controlling shallow subsurface dissolved reactive phosphorus concentration and loss kinetics from poorly drained saturated grassland soils. J. Environ. Qual. 52(2): 355–366. doi: 10.1002/jeq2.20442.

Soil Survey Staff. 2022. Kellogg Soil Survey Laboratory Methods Manual Version 6.0. U.S. Department of Agriculture Natural Resources Conservation Service, Lincoln, Nebraska.

Vehtari, A., J. Gabry, M. Magnusson, Y. Yao, P.-C. Bürkner, et al. 2023. loo: Efficient leave-one-out cross-validation and WAIC for Bayesian models. https://mc-stan.org/loo/.

Vehtari, A., A. Gelman, and J. Gabry. 2017. Practical Bayesian model evaluation using leave-one-out cross-validation and WAIC. Stat. Comput. 27(5): 1413–1432. doi: 10.1007/s11222-016-9696-4.

Wood, S.N. 2013. On p-values for smooth components of an extended generalized additive model. Biometrika 100(1): 221–228. doi: 10.1093/biomet/ass048.
